# Rational Design of Ti_3_C_2_T_*x*_ MXene Inks for Conductive, Transparent Films

**DOI:** 10.1021/acsnano.2c11180

**Published:** 2023-02-07

**Authors:** Tiezhu Guo, Di Zhou, Shungui Deng, Mohammad Jafarpour, Jonathan Avaro, Antonia Neels, Jakob Heier, Chuanfang Zhang

**Affiliations:** † Key Laboratory of Multifunctional Materials and Structures, Ministry of Education, School of Electronic Science and Engineering, Xi’an Jiaotong University, Xi’an710049, Shaanxi, China; ‡ Laboratory for Functional Polymers, Empa, Swiss Federal Laboratories for Materials Science and Technology, Überlandstrasse 129, CH-8600, Dübendorf, Switzerland; § Institute of Materials Science and Engineering, Ecole Polytechnique Federale de Lausanne (EPFL), Station 12, CH-1015Lausanne, Switzerland; ∥ Center for X-ray Analytics, Empa, Swiss Federal Laboratories for Materials Science and Technology, Lerchenfeldstrasse 5, CH-9014, St. Gallen, Switzerland; ⊥ Biomimetic Membranes and Textile, Empa, Swiss Federal Laboratories for Materials Science and Technology, Lerchenfeldstrasse 5, CH-9014, St. Gallen, Switzerland; # Center for X-ray Analytics, Empa, Swiss Federal Laboratories for Materials Science and Technology, Überlandstrasse 129, CH-8600, Dübendorf, Switzerland; ○ Department of Chemistry, University of Fribourg, Chemin du Musée 9, CH-1700, Fribourg, Switzerland; □ College of Materials Science & Engineering, Sichuan University, Chengdu, 610065, Sichuan, China

**Keywords:** Ti_3_C_2_T_*x*_ MXene, transparent conductive electrodes, percolation, blade coating, supercapacitors, Joule heaters

## Abstract

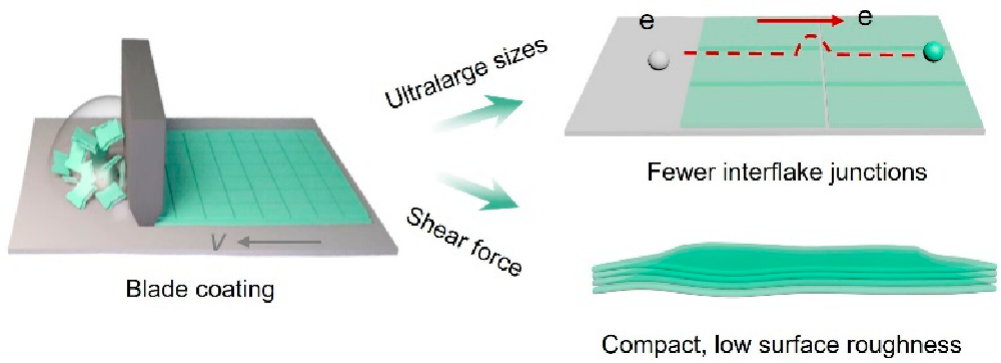

Transparent conductive electrodes (TCEs) with a high figure of merit (FOM_e_, defined as the ratio of transmittance to sheet resistance) are crucial for transparent electronic devices, such as touch screens, micro-supercapacitors, and transparent antennas. Two-dimensional (2D) titanium carbide (Ti_3_C_2_T_*x*_), known as MXene, possesses metallic conductivity and a hydrophilic surface, suggesting dispersion stability of MXenes in aqueous media allowing the fabrication of MXene-based TCEs by solution processing. However, achieving high FOM_e_ MXene TCEs has been hindered mainly due to the low intrinsic conductivity caused by percolation problems. Here, we have managed to resolve these problems by (1) using large-sized Ti_3_C_2_T_*x*_ flakes (∼12.2 μm) to reduce interflake resistance and (2) constructing compact microstructures by blade coating. Consequently, excellent optoelectronic properties have been achieved in the blade-coated Ti_3_C_2_T_*x*_ films, *i*.*e*., a DC conductivity of 19 325 S cm^–1^ at transmittances of 83.4% (≈6.7 nm) was obtained. We also demonstrate the applications of Ti_3_C_2_T_*x*_ TCEs in transparent Joule heaters and the field of supercapacitors, showing an outstanding Joule heating effect and high rate response, respectively, suggesting enormous potential applications in flexible, transparent electronic devices.

Transparent conductive electrodes (TCEs) are considered as a critical component in next-generation electronics, such as touch screens, liquid-crystal displays, transparent antennas, and other fields.^1−3^ For TCEs, films possessing low sheet resistances (*R*_s_) at high transparency (*T*) are highly desired. However, increasing *T* means thinning down the electrode, which inevitably decreases the number of conductive paths, leading to percolation problems and an increase in *R*_s_.^4^ At present, commercial TCEs are still dominated by indium tin oxide (ITO) due to its ultralow *R*_s_ at ultrahigh *T*.^1,4,5^ However, the fragile nature of ITO limits its applications in flexible transparent electronics. In other words, resilient TCEs with durable flexibility that withstand repeated deformation are in high demand. Ultrathin films based on low-dimensional conductive carbon materials (i.e., carbon nanotubes, graphene), polymers (*i*.*e*., PEDOT: PSS),^6−8^ metals (*e.g.*, metal grids, silver nanowires),^3,9−12^ and metal/nanosheet composites^13,14^ are promising candidates for TCEs. To meet the minimum standard for industrial applications, TCEs require a transparency *T* of at least 90% and a *R*_s_ not higher than 100 Ω sq^–1^, which corresponds to a minimum figure of merit (FOM_e_) > 35, according to eq 1,^15^
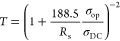
1where σ_op_ is the optical conductivity, σ_DC_ is the DC conductivity, and σ_DC_/σ_op_ is defined as the FOM_e_ value.

Although the transmittance loss of single-layer graphene is ∼2.3% (thickness ∼0.34 nm),^16,17^ undoped graphene TCEs are considered to possess fundamental limitations. In particular, the maximum FOM_e_ of liquid-exfoliated and reduced graphene oxide flakes is close to 2.^1^ A quasi-continuous graphene thin film grown by chemical vapor deposition (CVD) on the other hand displays an FOM_e_ up to 11.^1^ Despite the excellent optoelectric properties of metal grids, metallic meshes, and silver nanowires, the strict processing requirements for deposition from solution as well as the serious haze effect resulting from the disordered distribution of silver nanowires hinder their scalable industrial manufacturing and applications.^4^ One of the key challenges for the production of TCEs with desired optoelectronic properties is the quest for electrode materials that intrinsically possess high electrical conductivity and are free of percolation problems in the high-transparency region.

MXenes are a family of 2D transition metal carbides, nitrides, or carbonitrides, generally expressed as M_*n*+1_X_*n*_T_*x*_, where *n* = 1, 2, 3, and 4, M stands for a transition metal (Ti, Mo, Nb, V, *etc.*), X for C and/or N, and T_*x*_ for terminal groups (−O, −F, −OH, −Cl, *etc.*).^18,19^ The most studied Ti_3_C_2_T_*x*_ has been extensively researched in supercapacitors,^20,21^ electromagnetic-interference shielding,^22^ Na-ion batteries,^23^ Joule heating,^24^ and nitrogen fixation,^25^ to name just a few. Andrew *etal*. demonstrated that a single layer of Ti_3_C_2_T_*x*_ results in ∼3% loss in transmittance.^26^ At present, the disadvantages of MXene-based TCEs produced by various methods include low inherent conductivity,^26−30^ poorly adhering films,^31^ and high surface roughness.^32,33^ To the best of our knowledge, the prerequisite of high electrical conductivity at high transmittance in Ti_3_C_2_T_*x*_ is the formation of assembled ultrathin films possessing a continuous compact morphology with few junctions. Aside from the high conductivity, the compact, highly aligned nature also endows Ti_3_C_2_T_*x*_ films with high mechanical strength and toughness.^34,35^ For example, dopamine undergoes *in situ* polymerization on the surface of Ti_3_C_2_T_*x*_, resulting in an atomically thin polydopamine layer behaving as a binder and promoting the flake stacking/alignment.^36^ Cheng *etal*. achieved densification of Ti_3_C_2_T_*x*_ films and removal of voids by hydrogen bonding (*via* sodium carboxymethyl cellulose) and a covalent bond continuous bridging strategy (*via* borate ions), resulting in highly compact Ti_3_C_2_T_*x*_ films with high strength.^35^ However, the introduction of a nonconductive phase limits the possibilities to improve electrical conductivity. Alternatively, by utilizing shear forces to densify and align Ti_3_C_2_T_*x*_ flakes, highly compact MXene films can be fabricated. For instance, the centripetal force from spin coating shears the solution in a way that the flakes are distributed evenly across the flat substrates, leading to a higher degree of parallel alignment than in drop-coating.^26^ Compared to spin coating or dip coating, which typically lead to substantial material waste, blade coating of inks has the advantage of high material utilization and produces a higher shearing strength and anisotropy, as well as a higher degree of orientation when the blading speed is slow.^37,38^

Blade coating allows the production of films with different thickness (transparent to opaque) by simply adjusting the blade height and/or solution concentration, inducing the compact parallel arrangement of nanosheets by applying shear force.^39^ Zhang *etal*. demonstrated the scalable manufacturing of opaque Ti_3_C_2_T_*x*_ films, exhibiting high strength (∼570 MPa) and high electrical conductivity (∼15 100 S cm^–1^) by blade coating.^34^ However, reports on the fabrication of compact MXene TCEs with highly aligned flakes possessing excellent FOM_e_ are quite rare, to the best of our knowledge.

On top of morphology control, the electrical conductivity of Ti_3_C_2_T_*x*_ films can also be tuned by reducing the number of interflake junctions. In general, for films produced from large-sized 2D flakes, this number can be much lower compared to films fabricated from small-sized 2D flakes at a given thickness, leading to much higher electrical conductivity in the former.^40^ This highlights the importance of MXene flake size, in particular for TCEs which typically encounter percolation problems in the highly transparent region. In addition, the degree of Ti_3_C_2_T_*x*_ flake delamination and aggregation will affect the quantity of interflake tunnelling barriers. In other words, the preparation of uniform, predominantly single-layer Ti_3_C_2_T_*x*_ flakes with ultralarge lateral size is of significance when it comes to the realization of highly conductive Ti_3_C_2_T_*x*_-based TCEs.

Here, our work reports blade-coated Ti_3_C_2_T_*x*_ TCEs with compact flakes highly orientated along the substrates. The Ti_3_C_2_T_*x*_ TCEs exhibit a record-high FOM_e_ without observed obvious percolation problems. We believe several factors are responsible for the state-of-the-art optoelectronic properties: (1) the large-sized Ti_3_C_2_T_*x*_ flakes (12.2 μm) ensure a reduced quantity of interflake junctions for electron hopping, and (2) the shear force induced by blade coating guarantees a compact microstructure of large aspect ratio flakes, facilitating the electron transport among the transparent conductive films. We observe an excellent Joule-heating effect for the Ti_3_C_2_T_*x*_ TCEs and outstanding rate performance for solid-state transparent supercapacitors, suggesting the enormous potential of our blade-coated Ti_3_C_2_T_*x*_ TCEs in fabricating next-generation advanced transparent electronics.

## Results and Discussion

**Scheme 1:**
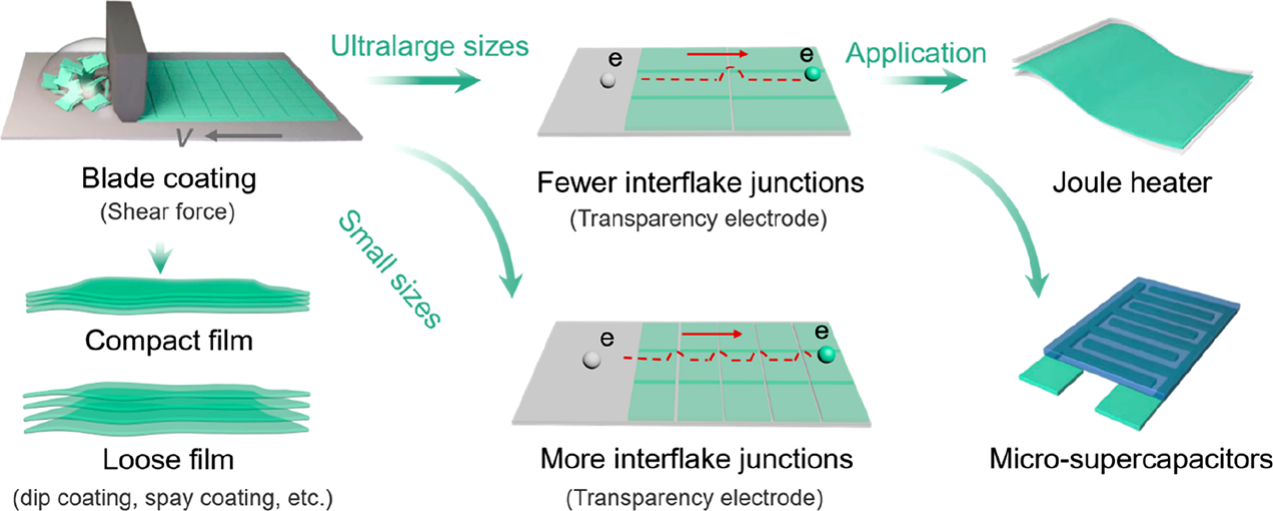
Scheme of the origin of ultrahigh conductivity in MXene TCEs through blade coating.

In this work, we employ blade coating of MXene aqueous inks to fabricate MXene TCEs. As demonstrated in Scheme 1, two aqueous inks were rationally designed enriched with large-sized flakes (lateral size up to ∼12.2 μm) and normal flakes (lateral size ∼1 μm), respectively. Detailed synthesis of MXene inks can be found in the Supporting Information. The blade-induced shear force and subsequent heat treatment enabled the Ti_3_C_2_T_*x*_ flakes to assemble into a compact architecture with large nanosheets orientated parallel to the substrate. The highest FOM_e_ value we could reach with this method was 29 with a film of a thickness of ∼7 nm showing an electrical conductivity of 21 750 S cm^–1^. To the best of our knowledge, this is the highest reported value to date for Ti_3_C_2_T_*x*_ TCEs, even higher than most opaque conductive films. The excellent optoelectronic properties of transparent MXene films ensure promising applications in transparent electronics requiring high conductivity and transmittance, such as transparent Joule heaters and solid-state micro-supercapacitors, as shown in Scheme 1.

**Figure 1:**
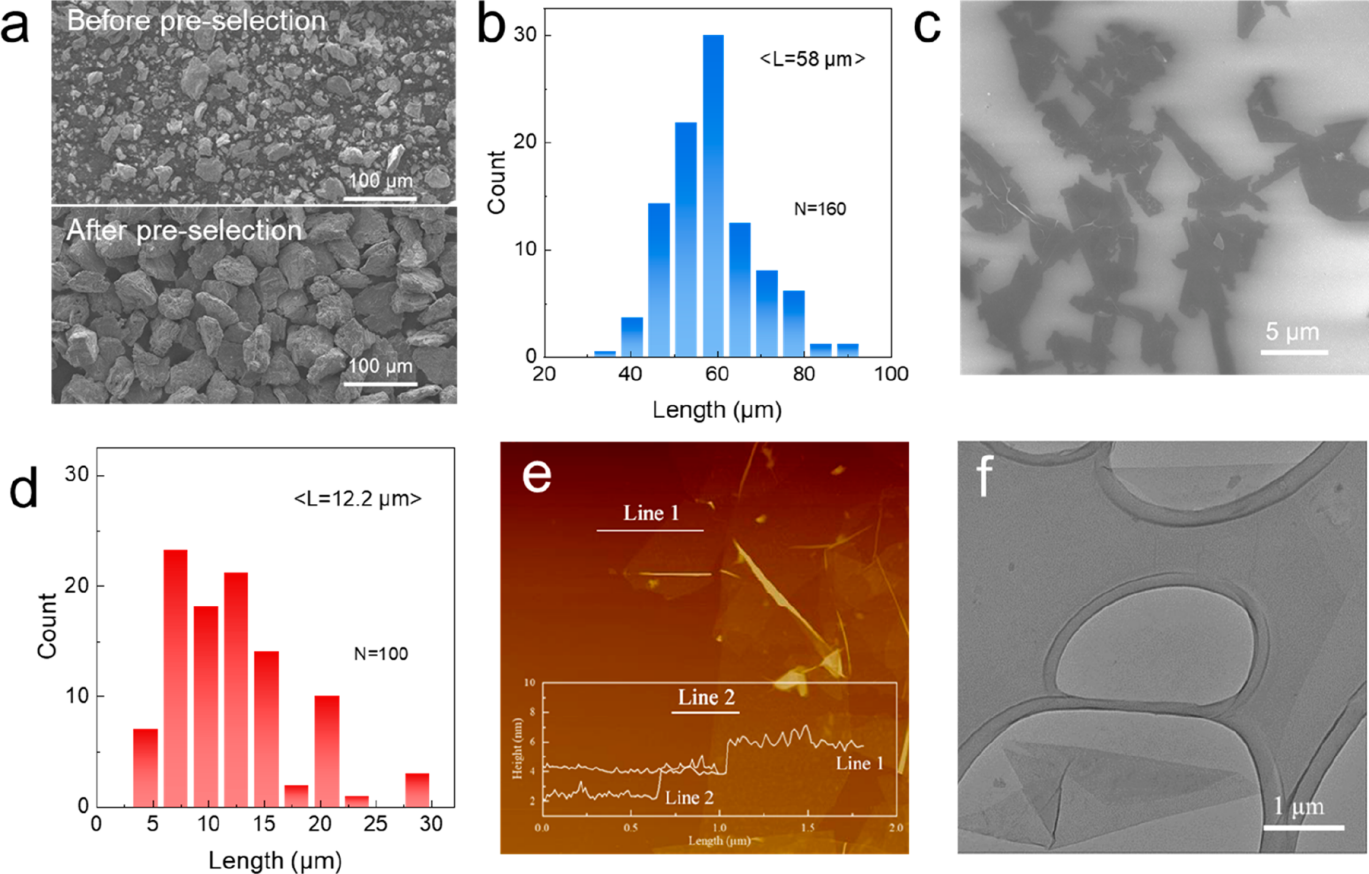
(a) SEM images of MAX phases before and after preselection. (b) Histogram of preselected MAX phases. SEM image (c) and the histogram (d) of Ti_3_C_2_T_*x*_ flakes. AFM (e) and TEM (f) images of Ti_3_C_2_T_*x*_ flakes.

We start by describing the formulation of high-quality MXene special inks. We preselected Ti_3_AlC_2_ particles of specific size from the as-received MAX phase by settling the Ti_3_AlC_2_ particles in water at different velocities based on Stokes’ law; experimental details are shown in the Supporting Information. The MAX phase with a mean particle size of 58 μm was collected, in sharp contrast to the as-received MAX powers where a broad size distribution is observed, as shown in Figure 1a,b. The preselected Ti_3_AlC_2_ was etched by the optimized MILD route (24 M LiF/9 M HCl) at 50 °C for 48 h as detailed in the Supporting Information. After multiple times of washing and subsequent density gradient centrifugation, viscous aqueous inks consisting of ultralarge Ti_3_C_2_T_*x*_ flakes with a narrow size distribution were obtained.

The successful removal of the Al element from the MAX phase and the successful preparation of Ti_3_C_2_T_*x*_ were proven by X-ray diffraction (XRD): the characteristic peak of the Ti_3_AlC_2_ MAX phase located at 39° disappears; instead, the peak centering at 6.6–6.8° is observed (Figure S1).^41,42^ The scanning electron microscope (SEM) image and the size histogram indicate a mean lateral size ⟨*L*⟩ of 12.2 μm in the delaminated MXene flakes, as shown in Figure 1c,d. The maximum lateral size of delaminated Ti_3_C_2_T_*x*_ is up to 30 μm, in sharp contrast to the sonicated Ti_3_C_2_T_*x*_ with an ⟨*L*⟩ of 1 μm (Figure S2). The thickness of the large Ti_3_C_2_T_*x*_ flakes is 1.2–1.5 nm (Figure 1e) based on the height profile from the atomic force microscopy (AFM) measurement, consistent with previous reports of single-layer Ti_3_C_2_T_*x*_ flakes.^43,44^ The predominantly large-sized single-layer Ti_3_C_2_T_*x*_ flake is transparent under the electron beam, possessing well-defined edges according to the transmission electron microscope (TEM) image shown in Figure 1f. The TEM image also indicates the high quality of as-delaminated flakes, as no pinholes or apparent defects are found on the flake surface (Figure S3). We believe the MXene clean surface coupled with their large lateral size is certainly beneficial for the excellent electronic conductivity, as will be discussed below.

**Figure 2:**
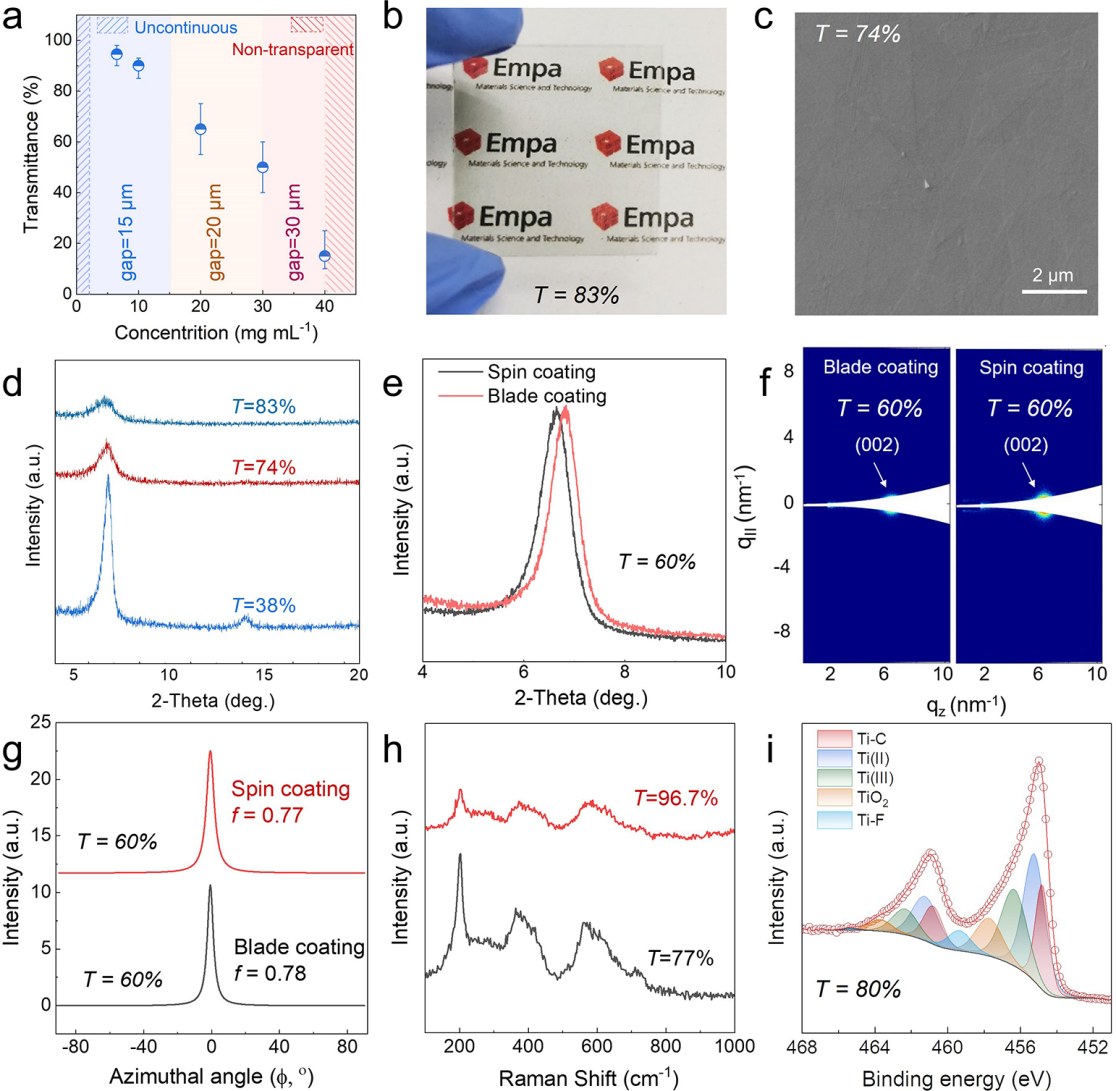
(a) Guiding map for MXene TCE fabrication. (b) Digital photograph of Ti_3_C_2_T_*x*_ films from large-sized flakes on glass (*T* = 83%). (c) Top-view SEM images of a blade-coated Ti_3_C_2_T_*x*_ film (*T* = 74%). (d) XRD patterns of various transparent films by blade coating. (e) XRD patterns of blade-coated and spin-coated Ti_3_C_2_T_*x*_ films (*T* = 60%) on glass. (f) GISAXS measurement of a Ti_3_C_2_T_*x*_ TCE (*T* = 60%). GISAXS detector image showing the (002) peak over *q*_*z*_. (g) Lorentzian fit of the azimuthal profile for the (002) peak used to determine the Herman’s degree of orientation. (h) Raman spectra of Ti_3_C_2_T_*x*_ films with different transparency. (i) XPS spectrum of a Ti_3_C_2_T_*x*_ film (*T* = 80%).

In general, the high quality (including the transparency, homogeneity, *etc.*) of the films fabricated through blade coating is governed by various factors such as blade height, ink concentration, blade speed, and substrates properties. To obtain highly transparent (*T* > 80%) and uniform conductive thin films, the concentration of Ti_3_C_2_T_*x*_ ink was kept at 2–10 mg mL^–1^, since a too diluted solution (<2 mg mL^–1^) leads to discontinuous thin films, while too concentrated (>30 mg mL^–1^) ink results in opaque (*T* < 20%) thin films. Aside from the ink concentration, the blade height was also adjusted to obtain transparent films with desired thickness or transparency, as illustrated in Figure 2a. After blade coating, vacuum annealing at 180 °C (glass substrate) was conducted to allow the capillary force densifying the as-deposited flakes, forming interconnected, compact Ti_3_C_2_T_*x*_ TCEs.^22^ A digital photograph of a typical Ti_3_C_2_T_*x*_ film (*T* = 83%) on glass substrate is shown in Figure 2b and Figure S4.

The SEM image (Figure 2c) showcases that the transparent film (*T* = 74%) possesses a flat smooth surface with large-sized flakes continuously and closely covering the glass substrate. We also compared SEM images of MXene TCEs from previously published works with our transparent film; it is clearly visible that our film does not show any of the sharp contrasts visible in other works (Figure S5). The cross-sectional FIB-SEM image (Figure S6) reveals a flat film thickness of ∼11 nm, consistent with AFM and profilometer measurements, which will be described later. To determine the alignment and compactness of the films, XRD was performed on transparent Ti_3_C_2_T_*x*_ films with different transmittances. As shown in Figure 2d, only (00*l*) reflection peaks are observed in the Ti_3_C_2_T_*x*_ thin film (*T* = 38%), indicating that Ti_3_C_2_T_*x*_ flakes are highly aligned in parallel to the glass substrate. Thinning down the film to *T* = 74% and *T* = 83% leads to broader (002) peaks with much lower peak intensity, in good agreement with the literature.^45^ Compared with the spin-coated film, the blade-coated film showcases a stronger (002) peak at 6.8° (spin-coating at 6.6°) and a smaller full-width-at-half-maximum of 0.56° (spin-coating, 0.61°, Figure 2e) at a given *T* = 60%, suggesting the Ti_3_C_2_T_*x*_ flakes are more compact (narrower interlamellar spacing) and better aligned (better crystallinity) in the blade-coated film than those of the spin-cast counterpart. The blade-coated films with large-sized or small-sized Ti_3_C_2_T_*x*_ flakes also showcase smaller interlayer spacings than filtered films (Figure S1).

To confirm the improved flake orientation in the blade-coated films, we employed grazing-incidence small-angle X-ray scattering (GISAXS) measurements. We compared the shape of the (002) reflection present at *q*_*z*_ ∼ 6.2 nm^–1^ (Figure 2f) and fitted its azimuthal profile with Lorentzian curves (Figure 2g). The degree of orientation was calculated using the Lorentzian curve and the Hermann orientation factor (*f*) as previously established for MXene flakes measured in transmission mode.^34^ The calculated *f* shows that the blade-coated film possesses a slightly higher value (0.78) than the spin-coated film (0.77). In addition, we also measured the orientation of the filtered opaque film (∼1 μm) to be *f* = 0.63 (Figure S7), similar to the values reported by Zhang *etal*.,^34^ which is significantly lower than in blade-coated transparent films. The Raman spectra of Ti_3_C_2_T_*x*_ films with different transmittance are shown in Figure 2h; all characteristic peaks agree well with previously recorded Raman spectra of Ti_3_C_2_T_*x*_,^44^ suggesting that no oxidation was observed in the films. This is further verified by X-ray photoelectron spectroscopy (XPS), as the chemical composition and structure of the transparent film (*T* = 80%) are quite similar to those of nontransparent fresh MXene films (Figure 2i).^46^

### Optoelectronic Properties of the Ti_3_C_2_T_*x*_ Thin Films

**Figure 3:**
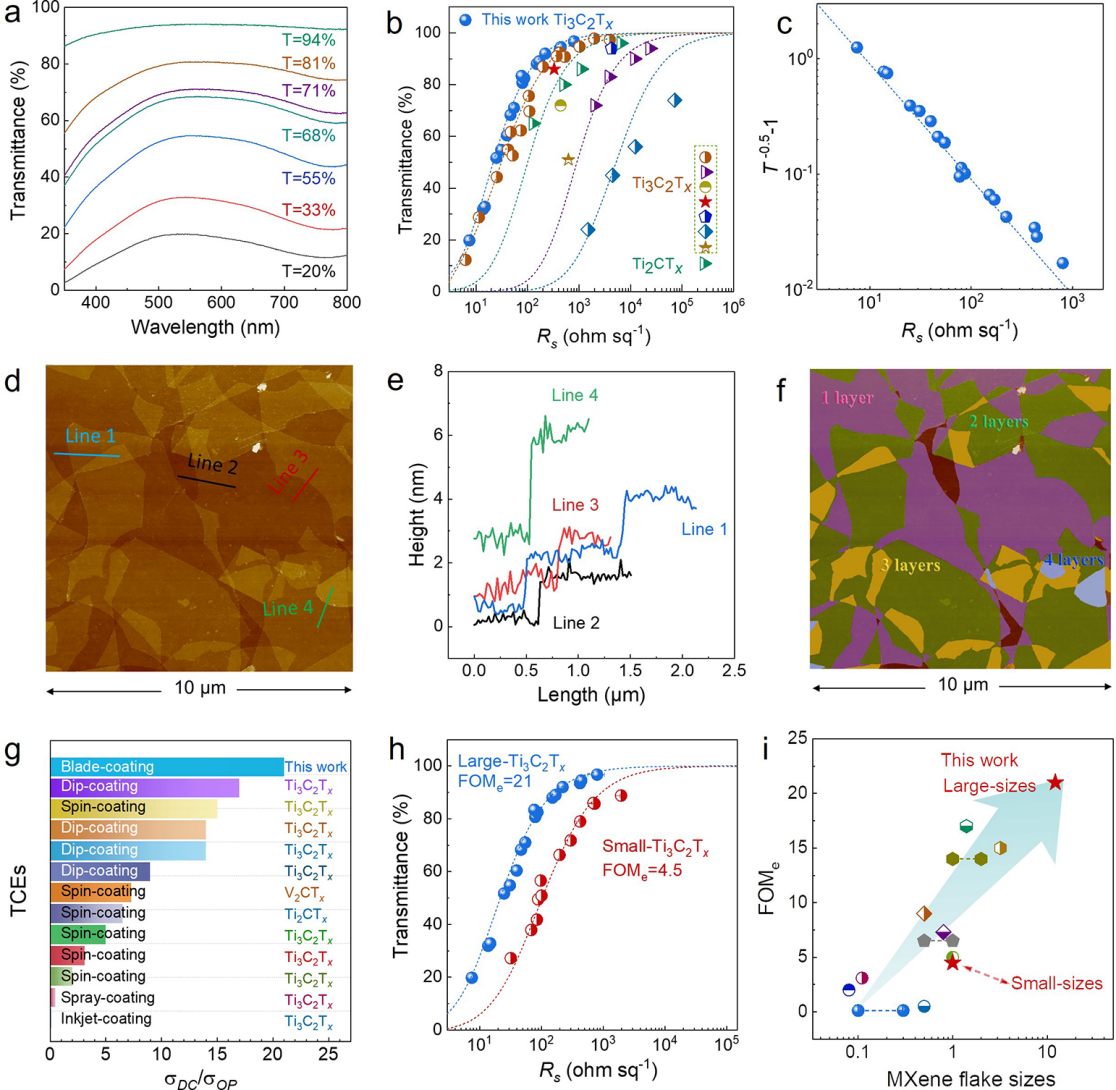
Optoelectronic properties of Ti_3_C_2_T_*x*_ thin films. (a) UV–vis spectra of films with various transmittance. (b) The relationship between transmittance (*T*) and sheet resistance (*R*_s_). Also included are fitting curves and (*T*, *R*_s_) from literature reports. (c) *T*^–0.5^ – 1 as a function of *R*_s_ to confirm the fitting value. (d) AFM image of a Ti_3_C_2_T_*x*_ film at *T* = 94% (MXene coverage 98%). (e) Height profiles of the different lines marked on (d). (f) Areas comprising different numbers of layers in (d) are shown with different colors. (g) Comparison of σ_DC_/σ_op_ (FOM_e_) in various transparent conductive MXene films; detailed values are presented in Table S2. (i) Relationship between FOM_e_ and MXene flake sizes.

As mentioned before, by adjusting factors such as ink concentration and/or blade height, films with different transmittances can be obtained at ease. The UV–vis spectra showcases characteristic peaks of Ti_3_C_2_T_*x*_ thin films in the visible region, with a broad peak in the thicker films (Figure 3a). We plotted the relationship between the sheet resistance (*R*_s_) and transmittance (*T*) at 550 nm of Ti_3_C_2_T_*x*_ thin films and fitted *T* as a function of *R*_s_ according to eq 1, as shown in Figure 3b. Quite interestingly, the highly transparent film (*T* = 96.7%) only exhibits an *R*_*s*_ of 800 Ω sq^–1^, significantly lower than that of other MXene-based TCEs (Ti_3_C_2_T_*x*_, Ti_2_CT_*x*_) with similar transmittance. Literature examples include films prepared by spin-coating Ti_2_CT_*x*_ (*T* = 96%, 6440 Ω sq^–1^),^33^ interfacial self-assembly Ti_3_C_2_T_*x*_ (*T* = 96.9%, 1623 Ω sq^–1^),^47^ and dip-coating Ti_3_C_2_T_*x*_ (*T* = 94%, 4300 Ω sq^–1^).^45^ The much reduced *R*_s_ suggests the advantage of our ink design as well as methodology in thin-film fabrication. In addition, previous transparent Ti_3_C_2_T_*x*_ MXene films based on spin-coating^28^ and inkjet-printing,^29^ have typically encountered percolation problems as *T* > 85%, best seen by the strongly deviated *R*_s_ from the fitting line.^1,4^ From fitting of the entire regime, we obtained the average FOM_e_ value of 21 (Figure 3b), the highest reported value to date for MXenes, to the best of our knowledge (*e.g.*, Ti_3_C_2_T_*x*_, Ti_2_CT_*x*_, V_2_CT_*x*_) thin films; detailed values are presented in Table S2. The best Ti_3_C_2_T_*x*_ film in this work exhibits an FOM_e_ value of 29. Interestingly, the sheet resistance of transparent Ti_3_C_2_T_*x*_ films is independent of film thickness, demonstrating a typical bulk-like conductivity. By fitting *T*^–0.5^ – 1 as a function of *R*_s_, we can further confirm that the bulk-like conductivity behavior applies to the transparent Ti_3_C_2_T_*x*_ films in the entire range of transmittance (20% < *T* < 94%, Figure 3c).^15^ More importantly, the (*R*_s_, *T*) data set of our TCEs follows the theoretical curve very well, even at high transmittance, indicative of the absence of notorious percolation problems. Specifically, we take the average value of the transmittance from five different regions as the transmittance of the TCEs, while three different surface regions were characterized by AFM, as shown in Figure 3d,e,f and Figure S8. At *T* = 94%, the large-sized single-layer flakes form continuous conductive pathways with a coverage of 98%, which effectively avoids the percolation problem. Clearly, the absence of percolation issues in our films guarantees their excellent optoelectronic properties. In addition, based on AFM images (Figure 3f), we could very accurately determine the average thickness of the *T* = 94% film. In the 10 × 10 μm area, the average film thickness is about 1.8 layers, which corresponds to about 2.2–2.7 nm (at 1.2–1.5 nm/layer). The details of the thickness calculation are presented in Table S1. In Figure 3g, Figure S9, and Table S2, 3 we show FOM_e_ values of reported MXene and undoped graphene films. Obviously, the FOM_e_ values of our MXene films have outperformed the solution-processed 2D inks; the latter also showcases non-bulk-like behavior due to percolation effects at high transparency. Such excellent optoelectronic properties in our blade-coated transparent MXene films can be fairly attributed but are not limited to the following reasons: (1) large-sized flakes suggest fewer junctions for electron hopping; (2) compact and highly aligned morphology ensures the efficient contact for the interflakes; and (3) the high-quality MXene flakes guarantees fast electron transport. To correlate performance to size, we fabricated MXene films using conventional small-size (average size of 1 μm) Ti_3_C_2_T_*x*_, revealing a relatively low FOM_e_ of 4.5 (Figure 3h), similar to the values reported previously.^27,33,48,49^ A systematic comparison among various MXene TCEs reported in the literature confirms this trend: the FOM_e_ increases as the flake size enlarges (Figure 3i). This means that for further improving the optoelectronic performance of MXene-based TCEs, one needs to substantially boost the flake sizes, until the FOM_e_ reaches the plateau (theoretical limit).

As film thickness largely governs sheet resistance, an accurate measurement/prediction of thickness is important. Here we measured the thickness of films by contact profilometry when the films are thicker than 20 nm and obtain the optical conductivity, σ_op_, using the following eq 2:^15^
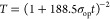
2where *t* is the thickness of films. We rearrange the above eq 2 to
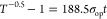
3

**Figure 4:**
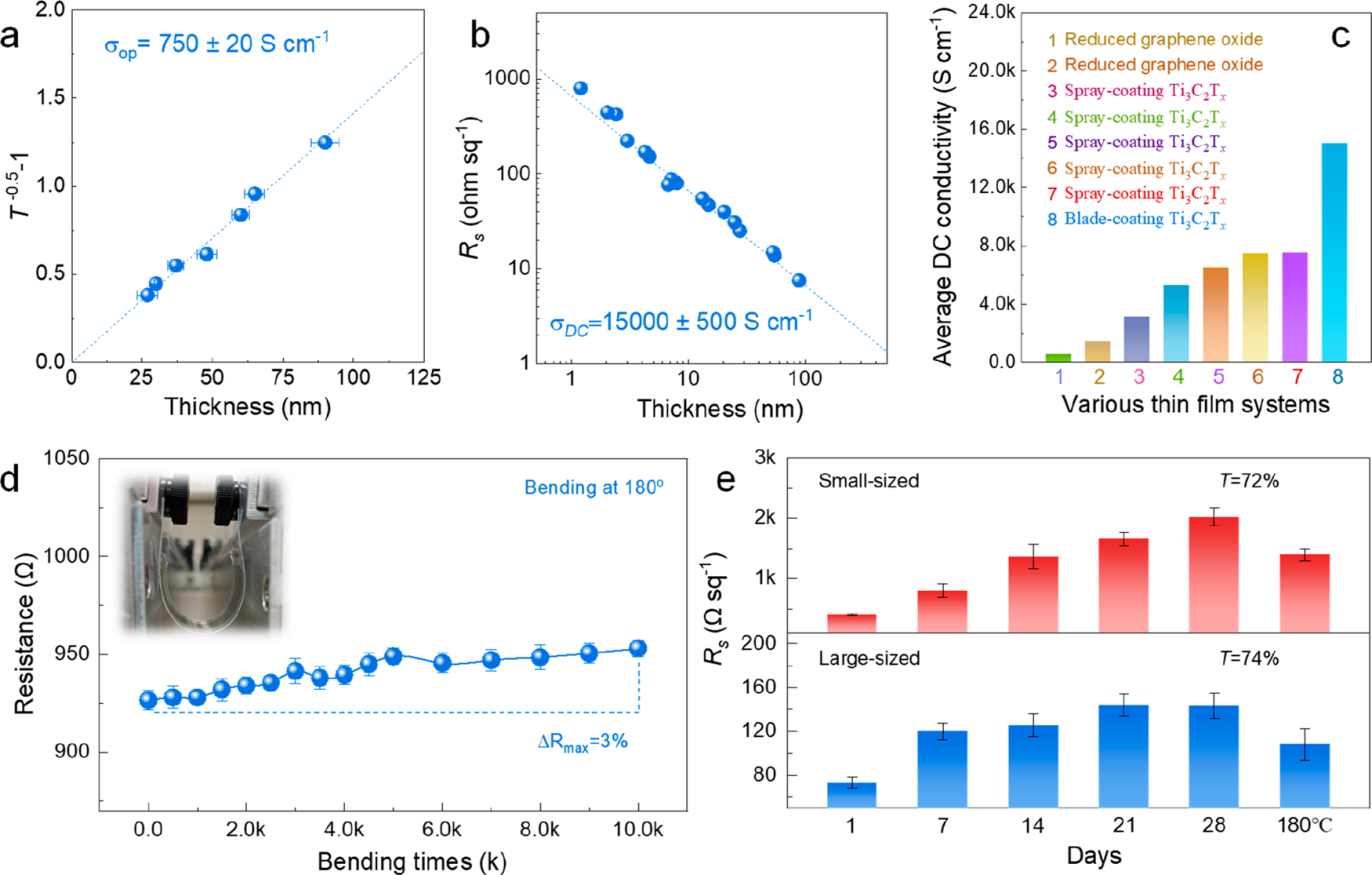
(a) *T*^–0.5^ – 1 as a function of thickness (*t*, >20 nm), to obtain the optical conductivity (σ_op_) of Ti_3_C_2_T_*x*_ thin films. (b) *R*_s_ as a function of *t* to extrapolate the average conductivity (σ_DC_) of Ti_3_C_2_T_*x*_ thin films. (c) Comparison of average electronic conductivity in various transparent conductive films. Detailed values are presented in Table S4. (d) Changes in resistance of TCEs after folding at 180° for 10 000 cycles. (e) *R*_s_ as a function of time under ambient conditions. The large-sized and small-sized flakes form a film with *T* = 74% and *T* = 72%, respectively.

Plotting *T*^–0.5^ – 1 as a function of *t* gives a linear curve with a slope equal to 188.5σ_op_. As such, we prepared a series of low-transmittance films from *T* = 52% to *T* = 20% on glass substrates to measure *T* and *t* through UV–vis and contact profilometry, respectively. The σ_op_ of Ti_3_C_2_T_*x*_ thin films is derived as 750 ± 20 S cm^–1^ (Figure 4a), which is slightly higher than that of Ti_3_C_2_T_*x*_ (520 S cm^–1^) from spin coating.^7^ σ_op_ is controlled by the intrinsic characteristics of Ti_3_C_2_T_*x*_ and the number of flakes per volume.^1^ We suggest that the variation in σ_op_ is due to film-to-film differences in morphological properties such as surface roughness. A lower transmissivity (corresponding to a higher σ_op_) in the blade-coated films than that of spin-coated films at a given thickness implies that blade-coated films are more compact with a larger total amount of aligned stacked flakes. This conclusion is also in line with the GISAXS result.

Based on eq 3 with a known σ_op_, any film’s thickness can be calculated from the measured *T*, as shown in Figure S10a. For example, the calculated film thickness of the *T* = 74% film (11.5 nm) is approximately the value extracted from the cross-sectional focused-ion-beam (FIB) SEM image (11 nm) and at *T* = 94% (2.2 nm) is approximately the average thickness of AFM characterization (2.2–2.7 nm). The results showcase that eq 3 is quite trustable in predicting the thickness of highly transparent MXene films. With the calculated thickness of all transparent films, their DC conductivity is then measured using eq 4:^7^
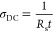
4

Impressively, the maximum DC conductivity of the transparent Ti_3_C_2_T_*x*_ film reaches 19 325 S cm^–1^; even the lowest value (9780 S cm^–1^) is quite similar to the reported best DC conductivity (9880 S cm^–1^)^7^ for Ti_3_C_2_T_*x*_ TCEs (Figure S10b,c). Importantly, these films are free from percolation problems, as when thinning down the films no precipitous conductivity decline is observed. By fitting *R*_s_ as a function of *t*, the average DC conductivity is derived (15 000 ± 500 S cm^–1^, Figure 4b), significantly higher than other TCEs based on either Ti_3_C_2_T_*x*_ or reduced graphene oxide (Figure 4c and Table S4). In addition, the inverse linear relationship between *R*_s_ and *t* also indicates that our MXene TCEs possess a bulk-like conductivity.^50^ Using PET substrates, the TCEs (*T* = 74%) demonstrated an increase in resistivity Δ*R*% of only 3% after 10 000 bending cycles at 180° (Figure 4d). Notably, the value is substantially lower than that of previously reported Ti_3_C_2_T_*x*_ TCEs on a PET substrate (increasing 20%, 1000 cycles).^32^Figure 4e showcases the ambient stability (*R*_s_ as a function of time) of prepared transparent films from large-sized and small-sized flakes, respectively. Obviously, the TCEs from large-sized flakes possess lower initial resistance and stronger ability to resist oxidation, and *R*_s_ increased from ∼73 to ∼143 Ω sq^–1^ after 28 days under ambient conditions. Following heat treatment (180 °C for 4 h) in an Ar atmosphere the values dropped down again to ∼108 Ω sq^–1^. This indicates that the increased resistance of TCEs under ambient conditions is caused by the synergistic effect of adsorption of water molecules and partial oxidation. As a result, we can conclude that moisture is an important factor affecting the **R*_*s*_* of the film under ambient atmosphere, and it is related to the initial resistance of the films.

### Joule-Heating Performance of Transparent Ti_3_C_2_T_*x*_ Films

**Figure 5:**
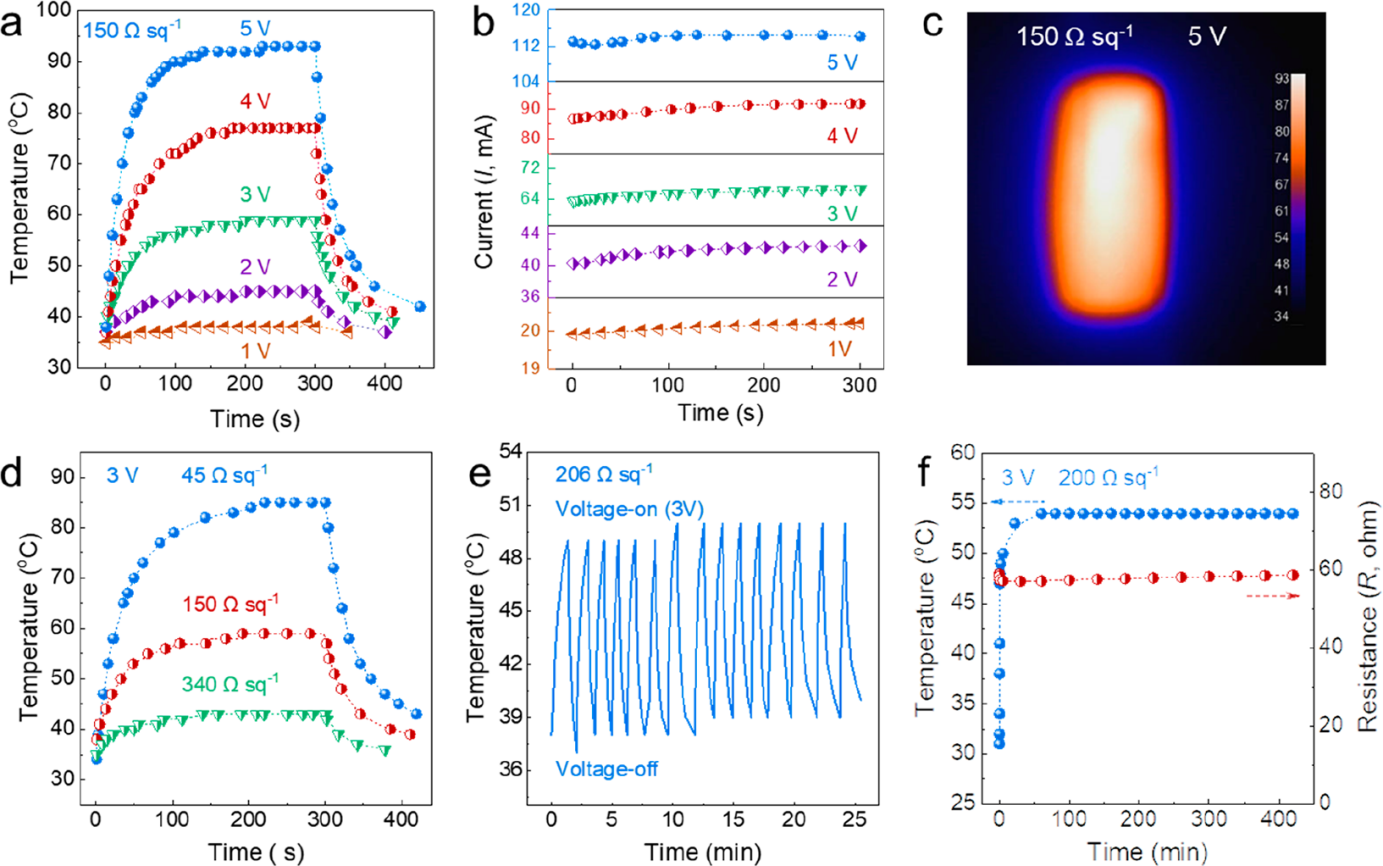
(a–c) thermal response of the transparent film (*T* = 87%, 150 Ω sq^–1^), including the temperature response (a), current response (b) at different onset voltage, and (c) infrared radiation image at 5 V. (d) The time-varying surface temperature of the transparent film with different *R*_s_ at 3 V. (e) Thermal response of repeated on–off at an applied voltage of 3 V. (f) Temperature and resistance change in the course of continuous operation for 7 h.

The excellent electrical conductivity enables tremendous potential of Ti_3_C_2_T_*x*_ TCEs in the field of thermoelectrics, such as healthcare and thermotherapy, through Joule heating.^11,24^ Fabricating transparent Joule heaters is of high significance, as it allows the direct visualizing of skin under the TCEs. Nevertheless, preparing high-performance Joule heaters with quick response time and low onset voltage is still quite challenging. This is because highly transparent films typically encounter high sheet resistance, leading to a high onset voltage to reach the desired temperature. Here the excellent optoelectronic properties in the as-fabricated MXene TCEs may suggest their promising application for advanced Joule heaters. Several transparent Ti_3_C_2_T_*x*_ TCEs with *R*_s_ from 45 Ω sq^–1^ (∼*T* = 67%) to 340 Ω sq^–1^ (∼*T* = 93%) were selected for Joule-heating analysis. As shown in Figure 5a, the temperature–time curves showcase the saturation temperature of the film with *R*_s_ = 150 Ω sq^–1^ (∼*T* = 87%) gradually increasing from 39, to 45, 59, 77, and 93 °C as the applied voltage is increased from 1 V, to 2, 3, 4, and 5 V, respectively. According to Joule’s law, , where *Q* is the heat generated by the Joule effect, *V* is the applied voltage, *R* is the resistance of the electrode, and *t* is the time. Obviously, the heat generated is related to the applied voltage and resistance. Here, the response time from room temperature to near saturation temperature is about 86 s at 5 V, which is significantly lower than that of opaque Ti_3_C_2_T_*x*_ coating commercial cellulose fabric (16 s@100 °C, 6 V),^24^ Ag micromesh/Ti_3_C_2_T_*x*_ (28 s@99 °C, *T* = 80%, 1.2 V),^51^ and PVDF-AgNW/Ti_3_C_2_T_*x*_ (25 s@77 °C, 2.5 V),^52^ depending on the resistance of the electrode. However, for highly transparent electrodes, this is desirable and acceptable. After applying a voltage to the Ti_3_C_2_T_*x*_ electrodes, the corresponding current values gradually increase to a constant value (Figure 5b), indicating that applying a voltage improves the conductivity and could be explained as the removal of surface species on the Ti_3_C_2_T_*x*_ TCEs.^53^ The infrared radiation (IR) image of *R*_s_ = 150 Ω sq^–1^ at 5 V is shown in Figure 5c, indicating the Ti_3_C_2_T_*x*_ thin film possesses good thermal uniformity.

The sheet resistance of the transparent electrode is a crucial factor affecting the surface saturation temperature; low sheet resistance can convert more heat at a given applied voltage. We investigated the electrothermal properties of Ti_3_C_2_T_*x*_ films with different sheet resistance. For the *T* = 67% film with *R*_s_ = 45 Ω sq^–1^, the saturated temperature reaches 86 and 115 °C under the applied voltage of 3 V (Figure 5d) and 4 V (Figure S11a,b,c), while the corresponding current values decrease above 86 °C, indicating continuous deterioration of service life, despite the constant temperature detected by IR thermometer. This phenomenon is not observed in the transparent electrode with *R*_s_ = 150 Ω sq^–1^ (no attenuation), which is mainly due to the fact that the lower sheet resistance electrode possesses a higher current value (45 Ω sq^–1^, ∼300 mA, 4 V) than the high sheet resistance electrode (150 Ω sq^–1^, 113 mA, 5 V). Although the electrode of 150 Ω sq^–1^ reached 93 °C at 5 V, it can still maintain a constant current within a certain time frame. Consequently, it is necessary to balance the relationship between applied voltage, response saturation temperature, and lifespan. For the highly transparent film (*R*_s_ = 340 Ω sq^–1^, *T* = 93%), an onset voltage results in a saturated temperature of 43 °C (Figure 5d) and 62 °C (Figure S12d,e,f) under 3 and 5 V, respectively. Moreover, the Joule-heating effect of the Ti_3_C_2_T_*x*_ thin film (206 Ω sq^–1^) demonstrated excellent cyclic on–off thermal response at the applied voltage of 3 V, as shown in Figure 5e. The saturation temperature can be maintained at 50 ± 1 °C during 15 cycles without a decline in thermal response time. The Joule heater durability of the MXene TCE (200 Ω sq^–1^) was evaluated by measuring the temperature changes with time at 3 V, as shown in Figure 5f. After 7 h of continuous operation, the maximum temperature remains constant with negligible resistance changes, demonstrating the excellent durability of our MXene TCE as transparent Joule heater.

### Electrochemical Performance of Transparent Ti_3_C_2_T_*x*_ Films

**Figure 6:**
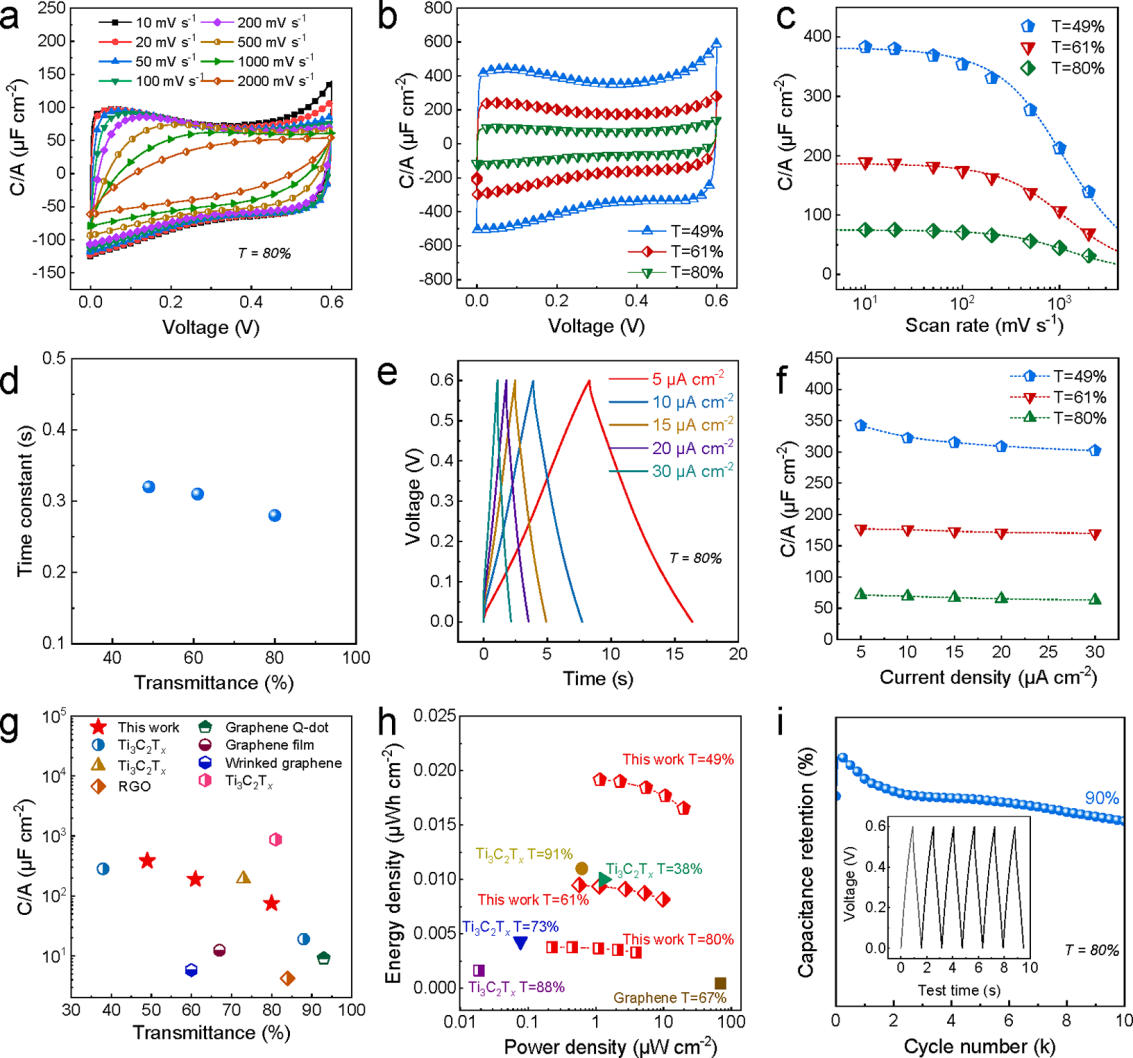
Electrochemical characterization of a transparent Ti_3_C_2_T_*x*_ MSC. (a) Normalized CV curves at various scan rates of the MSC device with *T* = 80%. (b) CV curves with different transparency at 10 mV s^–1^. (c) Measured areal capacitance obtained from CV curves. The dashed lines represent the capacitance fitting value according to eq 5. (d) The obtained time constant (Figure 6c), versus transmittance. (e) GCD curves at different current densities of devices, *T* = 80%. (f) Measured areal capacitance obtained from GCD curves of various transparency symmetric micro-supercapacitors. (g) Areal capacitance versus transmittance and comparison to other transparent supercapacitors. Detailed values are presented in Table S5. (h) Ragone plots of symmetric supercapacitors using different transparent electrodes and comparison to other transparent supercapacitors. Detailed values are presented in Table S6. (i) Long-term cycling of the transparent micro-supercapacitor (*T* = 80%). Note, the transmittance of the electrode before laser engraving is defined as the transmittance of the MSC, while the transmittance of the interdigitated MSC after laser engraving is much higher than that of the electrode before engraving; furthermore the transmittance of the MSC depends on the interdigitated finger gap of the MSC.

Finally, we investigated the electrochemical charge storage properties of symmetric micro-supercapacitors (MSCs, interdigitated finger gap ∼260 μm, Figure S12) based on Ti_3_C_2_T_*x*_ TCEs. The normalized cyclic voltammograms (CVs) of MSC were studied from 10 mV s^–1^ to 2000 mV s^–1^ as shown in Figure 6a and Figure S13. The MSC with a device transmittance of *T* = 80% exhibits a quasi-rectangular shape below 500 mV s^–1^ and favorable capacitance even at 2000 mV s^–1^ (maintaining 42% of initial capacitance at 10 mV s^–1^), indicating excellent rate performance. A quick CV comparison of MSCs with *T* = 80%, 61%, and 49% at 10 mV s^–1^ indicates that thicker films lead to higher capacitances, as seen in the gradually enlarged encircled area under the CV curves (Figure 6b). This electrochemical behavior is similar to that of opaque Ti_3_C_2_T_*x*_ based supercapacitors,^20,21^ indicating that our films have a good application prospect in the field of transparent energy storage devices. However, the thicker film showcases a sluggish time response upon voltage reversal at 100 mV s^–1^ compared to 10 mV s^–1^, coupled with the broad peak position (0–0.15 V) shifting to higher voltage (Figure S14). The areal capacitances of Ti_3_C_2_T_*x*_ at different scan rates were calculated from CV curves (Figure 6c) and were fitted based on eq 5,^15^
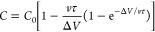
5where τ = *R*_ESR_*C* is the time constant, *v* is the scan rate,  is the voltage window (0.6 V), and *C*_0_ is the intrinsic, rate-independent areal capacitance (no electronic and/or ionic transport limitations during charge–discharge). The time constant extracted from the fits is used to characterize the migration kinetics of ions; a smaller time constant indicates faster ion migration. The *T* = 80% MSC exhibits 75.2 μF cm^–2^ at 10 mV s^–1^ from the experimental measurement; the value is slightly higher than that of graphene transparent films (*T* = 67%@12.4 μF cm^–2^, *T* = 84%@4.2 μF cm^–2^).^54,55^ However, it is substantially lower than the values for the small-size Ti_3_C_2_T_*x*_-based MSC at similar transparency (*T* = 81%@870 μF cm^–2^).^7^ The *T* = 61% and *T* = 49% MSCs exhibit 189.5 and 383.2 μF cm^–2^ at 10 mV s^–1^, respectively. This highlights the importance of flake size engineering when the TCEs are used for transparent energy storage devices. The lower capacitance at higher rates for thicker film-based MSCs can be explained with the larger time constant (Figure 6d). The galvanostatic charge–discharge (GCD) curves of MSCs are symmetric and triangular, indicating typical capacitive-like behavior (Figure 6e). The capacitance values derived from GCD curves are shown in Figure 6f. In addition, as the current density increases from 5 μA cm^–2^ to 30 μA cm^–2^, a high capacitance of 88% was achieved, suggesting very good rate handling properties in our transparent solid-state MXene MSCs. Figure 6g and Table S5 compare the areal capacitance of this work with other reported transparent MSC devices. The energy density and power density of Ti_3_C_2_T_*x*_ MSC with different transmittances were further calculated and compared with previous transparent MSC devices (Figure 6h, Table S6). The maximum energy density of our MXene MSC (*T* = 80%) reaches 0.004 μWh cm^–2^ (at a power density of 0.23 μW cm^–2^), which has greatly outperformed that of other MSCs, *i*.*e*., graphene (0.00047 μWh cm^–2^, *T* = 67%).^55^ The transparent MXene MSC also showcases an outstanding cycling performance, retaining 90% of the initial capacitance after 10 000 cycles (Figure 6i). These outstanding charge storage properties indicate the promising future of MXene MSCs for scalable fabrication of solid-state, advanced transparent MSCs to power microelectronics.

## Conclusion

In summary, we have fabricated high-performance Ti_3_C_2_T_*x*_ TCEs with compact microstructures, well orientated by room-temperature blade-shearing of the high-quality (predominantly single-layer, clean surface, narrow size distribution) additive-free MXene aqueous ink. The ultralarge, high-aspect-ratio flakes are forced to align, forming well-stacked, interconnected conductive paths without notorious percolation issues. Consequently, blade-coated MXene films achieved ultrahigh DC conductivity up to 19 325 S cm^–1^ at transmittances of 83.4% (≈6.7 nm). Such advanced optoelectronic properties ensure an excellent Joule-heating effect (thermal homogeneousness, durability, low onset voltage) and high rate response in transparent Joule heaters and transparent MSCs, respectively. The excellent optoelectric, electrothermal, and electrochemical properties suggest potential applications in flexible, transparent electronic devices. It is expected that the optoelectronic performance can be further improved by further boosting the flake sizes, optimizing the synthesis of MXene, regulating the surface chemistry, or/and the doping of pure Ti_3_C_2_T_*x*_, to name just a few.

## Methods

### Purification and Size Selection of Ti_3_AlC_2_ MAX Phase

The as-received Ti_3_AlC_2_ particles (10 g) were stirred continuously in 9 M HCl (35 mL) for 12 h and then dried for future use. The large-sized MAX phase was obtained following a modified version of a previously reported method.^34^ The purified MAX phase was dispersed in 50 mL of deionized water (centrifuge tube, height = 10 cm) and shaken evenly. After the dispersion was kept stationary for 82 s, the upper suspension was removed to separate the MAX phase particles larger than 25 μm, repeated three times. The large-size MAX sediment was collected and dried for further use. The standing time for the dispersion was selected based on the terminal velocity (, m s^–1^) according to eq 6:
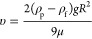
6where *g* is the gravitational acceleration (m s^–2^), *R* is the radius of the spherical particle, ρ_p_ is the mass density of the Ti_3_AlC_2_ MAX phase particles (∼4.2 × 10^3^ kg m^–3^), ρ_f_ is the mass density of water (10^3^ kg m^–3^), and μ is the dynamic viscosity (Pa s or kg m^–1^ s^–1^) of water (8.90 × 10^–4^ Pa s at ∼25 °C).

### Synthesis of Delaminated Large-Sized Ti_3_C_2_T_*x*_

The large-sized Ti_3_C_2_T_*x*_ was synthesized using a modified minimally intensive layer delamination method (MILD). Compared to the small-sized Ti_3_AlC_2_ MAX phase, the LiF/MAX ratio and etch time were optimized. Specifically, 2.4 g of LiF was slowly added into 9 M HCl (30 mL) and stirred continuously for 10 min in an oil bath of 50 °C. In the following 0.75 g of MAX phase was added to the above-mentioned solution in batches. After 48 h, the etched sediment was washed with deionized water and centrifuged at 1500 rcf for 5 min, and the supernatant was decanted. The process was repeated several times until the supernatant appeared dark-green and the pH value of the supernatant approached ∼6. Then, 40 mL of deionized water was added to the precipitate under manual shaking until the precipitate is completely redispersed (the suspension is centrifuged at 1500 rcf for 5 min, redispersed by manual shaking, without decanting the upper suspension, and repeated several times to improve the yield of Ti_3_C_2_T_*x*_ flakes). Furthermore, the samples were placed on a vortex machine and shaken for 30 min to improve the yield. After that, the suspension was centrifuged at 1500 rcf for 30 min. The upper black-green suspension was collected and labeled as a Ti_3_C_2_T_*x*_ colloidal solution with wider size distribution. Further, the colloidal solution was centrifuged at 3000 rcf for 15 min (the suspension contains relatively small nanosheets, the sediment was redispersed with deionized water by vigorous shaking), repeated twice. The sediment was collected, which now contains predominantly ultralarge-sized single-layer Ti_3_C_2_T_*x*_ flakes and labeled as ultralarge-sized Ti_3_C_2_T_*x*_ flakes and placed in a refrigerator at 4 °C. Finally, the precipitate was diluted to a specific concentration for blade-coating. The concentration of the delaminated Ti_3_C_2_T_*x*_ was determined by vacuum filtration with a Celgard membrane (Celgard 3501, PP coated, USA) and a known volume of the Ti_3_C_2_T_*x*_ aqueous solution.

### Synthesis of Small-Sized Ti_3_C_2_T_*x*_

We prepared small-sized Ti_3_C_2_T_*x*_ flakes by ultrasonification. Specifically, 0.8 g of LiF was slowly added into 9 M HCl (10 mL) and stirred continuously for 10 min in an oil bath of 35 °C. In the following 0.5 g of Ti_3_AlC_2_ MAX phase was added to the above solution in batches. After 24 h, the etched sediment was washed with deionized water and centrifuged at 1500 rcf for 5 min, and the supernatant was decanted. The process was repeated several times until the supernatant appeared dark-green and the pH value of the supernatant approached ∼6. Then, 100 mL of deionized water was added to the precipitate, and manual shaking was applied until the precipitates were completely redispersed. The suspension was continuously sonicated under an Ar atmosphere (after being degassed for 10 min) in an ice bath for 30 min, 35 kHz. The dispersion was centrifuged at 1500 rcf for 30 min, and the upper suspension was collected and labeled as small-sized Ti_3_C_2_T_*x*_ flakes.

### Production of Transparent, Conductive Thin Films

The transparent, conductive films were fabricated by blade-coating on glass and PET substrates in a class 5 clean-room. First, glass substrates were cleaned with a soap solution, ethanol, and deionized water, sequentially. After that, all the glass substrates and PET were plasma cleaned (Diener Plasma surface technology) for 2 min under vacuum (0.47 mbar), to obtain a hydrophilic surface. The conductive Ti_3_C_2_T_*x*_ films with various transparency were fabricated by adjusting the blade height and the concentration of the Ti_3_C_2_T_*x*_ solution. The films of transmittance >80% were prepared using a 2–10 mg mL^–1^ dispersion of large-sized Ti_3_C_2_T_*x*_ flakes at gap sizes of 15 μm, and the blade moving speed was controlled at 30–40 mm s^–1^. The films of transmittance of 40–80% were prepared using approximately 10–30 mg mL^–1^ dispersions at a gap height of 20 μm. The overconcentration of Ti_3_C_2_T_*x*_ solution is not conducive for the transparent and homogeneous films. The transparent films were transferred to a glovebox and annealed at 180 °C for 4 h (glass substrate) to eliminate the trapped water between the flakes. All samples were stored in the glovebox for future use.

### Morphology Characterization

SEM and TEM imaging of Ti_3_C_2_T_*x*_ flakes were performed on a NanoSEM 230 and a JEOL 2200 FS using an accelerating voltage of 200 kV. XRD patterns of Ti_3_C_2_T_*x*_ films were obtained using an X’Pert Pro with Cu Kα radiation (λ = 0.15406 nm). Atomic force microscopy measurements were performed on a Bruker ICON3 in the peak force scanasyst mode. Raman spectra of transparent films were measured using a Renishaw Raman microscope (633 nm).

GISAXS spectra of thin films were collected on a Bruker NanoStar (Bruker AXS GmbH, Karlsruhe, Germany) at the Center for X-ray Analytics at EMPA St. Gallen. The instrument was equipped with a pinhole collimation system, allowing a beam size at a sample position of about 400 μm in diameter. X-ray generation was sustained with a microfocused X-ray Cu source (wavelength Cu Kα = 1.5406 Å), and scattering patterns were recorded on a 2D MikroGap technology-based detector (VÅNTEC-2000 2D with 2048 × 2048 pixels and 68 × 68 μm pixel size) along with a standard beam stop. In the SAXS experimental configuration, the sample to detector distance was set at 27 cm and was further calibrated with a silver behenate powder standard, resulting in a scattering vector modulus *q* covering a range between 0.11 and 10.34 nm^–1^. To limit air scattering, the scattering patterns were recorded at room temperature under moderate vacuum conditions (10^–2^ mbar). Scattering of each sample was recorded over an integration time of 100 s, acquiring a good signal-to-noise ratio data set. Intensity data have been flat field, efficiency and spatially corrected, accounting for variations in the detector’s pixel-to-pixel sensitivity and geometry. Azimuthal profiles of (002) were recorded based on peak maxima *q*_*z*_ ≈ 6.2 nm^–1^ and fitted with a Lorentzian distribution curve. Herman’s orientation factor (*f*) calculation was used to describe the degree of orientation of the MXene flakes relative to the surface of the glass using the following eqs 7 and 8:^34,56^
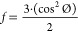
7where  is the mean-square cosine calculated from the scattered intensity *I*() integrated over the azimuthal angle following
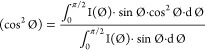
8The Herman’s orientation factor (*f*) values range from −0.5 ≤ *f* ≤ 1.0. If *f* is equal to −0.5, the orientation of flakes is perpendicular to the reference, *i*.*e*., flow, direction (φ = 90°), if *f* is equal to 1, the orientation is parallel to the reference direction (φ = 0°), and if *f* is equal to 0, a random orientation is present.

### Optoelectronic Property Measurements

The transmittance of the transparent electrode was measured by a UV–vis spectrophotometer (Varian Cary 50) in the wavelength range 350–800 nm. The transmittance at 550 nm is labeled as the transmittance of the films. The sheet resistance (*R*_s_) was tested with a four-point probe (Jandel, model RM3-AR), and the values were obtained by averaging 10 different regions for each sample. The thickness measurement of the low-transparency conductive films was performed by a programmable surface profiler (DEKTAK 6M), and the values were obtained by averaging in five different regions for each sample.

### Joule-Heating Characterization

The transparent heaters were powered with a DC voltage source (Keithley 2400). Temperature and thermal images were recorded by an IR camera (Seekthermal). The sample size was about 0.5 × 1.5 cm, and the height between the sample and the IR camera was about 7 cm.

### Electrochemical Characterization

PVA/H2SO4 hydrogel was obtained based on our previously reported method.^7^ Typically, 1 g of poly(vinyl alcohol) (PVA) powder was added to 10 mL of deionized water. Then the suspension was stirred vigorously at 85 °C until the solution became clear. After cooling down, 3 g of concentrated H_2_SO_4_ (97 wt.%) was added to the above solution, followed by another 1 h of vigorous stirring at room temperature. The transparent films were blade-coated onto a glass substrate. Consecutively the film was patterned by laser scribing (TruMark Station 5000) to prepare micro-supercapacitors. The laser scribing pattern was designed by TRUMPF software (interdigitated finger gap ∼260 μm). The electrochemical performance of the prepared micro-supercapacitors was evaluated by CV and GCD on a VMP3 potentiostat (BioLogic, France). The CVs of transparent supercapacitors were performed at 10–2000 mV s^–1^, and the GCD was performed at 2–30 μA cm^–2^ in a voltage window of 0.6 V. The area capacitance of the supercapacitor device was calculated from the third cycle of each CV test by eq 9:
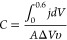
9where *C* is the area capacitance of supercapacitors (μA cm^–2^), *j* is the current (mA), Δ*V* is the voltage window (0.6 V), *A* is the geometric area of the MSC (0.38 cm^2^), and  is the scan rate (mV s^–1^). We also calculated the area capacitance of the supercapacitor device through the third cycle of each GCD curve according to eq 10:
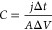
10where Δ*V* is the effective voltage window excluding the IR drop. Δ*t* is the discharge time.

## Supporting Information Available

Schematic illustration of the synthesis process, XRD spectra of MXene films, SEM and TEM images of MXene flakes, digital photography of MXene films on glass and PET, SEM images from previously published works on transparent MXene films, cross-sectional SEM image and GISAXS detector image of MXene films, methodology to extract average film thickness of ultrathin films, electrochemical characterization of films, relationship between Ti_3_C_2_T_*x*_ films thickness and optoelectronic properties, electrothermal properties of Ti_3_C_2_T_*x*_ films (PDF)
