# Supplementary material for: Rational Design of Ti3C2Tx MXene Inks for Conductive, Transparent Films
Source: ACS Nano. 2023 Feb 7;17(4):3737–49. doi: 10.1021/acsnano.2c11180 (PMC9979651; doi:10.1021/acsnano.2c11180)
Supplement: Supplementary file 1 — nn2c11180_si_001.pdf [file nn2c11180_si_001.pdf]

# Supporting information

## Rational Design of $Ti_3C_2T_x$ MXene Inks for Conductive, Transparent Films

Tiezhu Guo[1,2], Di Zhou[1*], Shungui Deng[2,3], Mohammad Jafarpour[2,3], Jonathan Avaro[4,5], Antonia Neels[6,7], Jakob Heier[2*], Chuanfang (John) Zhang[8*]

1 Key Laboratory of Multifunctional Materials and Structures, Ministry of Education, School of Electronic Science and Engineering, Xi'an Jiaotong University, Xi'an 710049, Shaanxi, China

2 Laboratory for Functional Polymers, Empa, Swiss Federal Laboratories for Materials Science and Technology, Überlandstrasse 129, CH-8600, Dübendorf, Switzerland

3 Institute of Materials Science and Engineering, Ecole Polytechnique Federale de Lausanne (EPFL), Station 12, CH-1015 Lausanne, Switzerland

4 Center for X-ray Analytics, Empa, Swiss Federal Laboratories for Materials Science and Technology, Lerchenfeldstrasse 5, CH-9014, St. Gallen, Switzerland.

5 Biomimetic Membranes and Textile, Empa, Swiss Federal Laboratories for Materials Science and Technology, Lerchenfeldstrasse 5, St. Gallen CH-9014, Switzerland.

6 Center for X-ray Analytics, Empa, Swiss Federal Laboratories for Materials Science and Technology, Überlandstrasse 129, CH-8600, Dübendorf, Switzerland.

7 Department of Chemistry, University of Fribourg, Chemin du Musée 9, CH-1700, Fribourg, Switzerland

8 College of Materials Science & Engineering, Sichuan University, Chengdu, 610065, Sichuan, China

[1]Corresponding author (zhoudi1220@mail.xjtu.edu.cn)

[2]Corresponding author (Jakob.Heier@empa.ch)

[3]Corresponding author (chuanfang.zhang@scu.edu.cn)

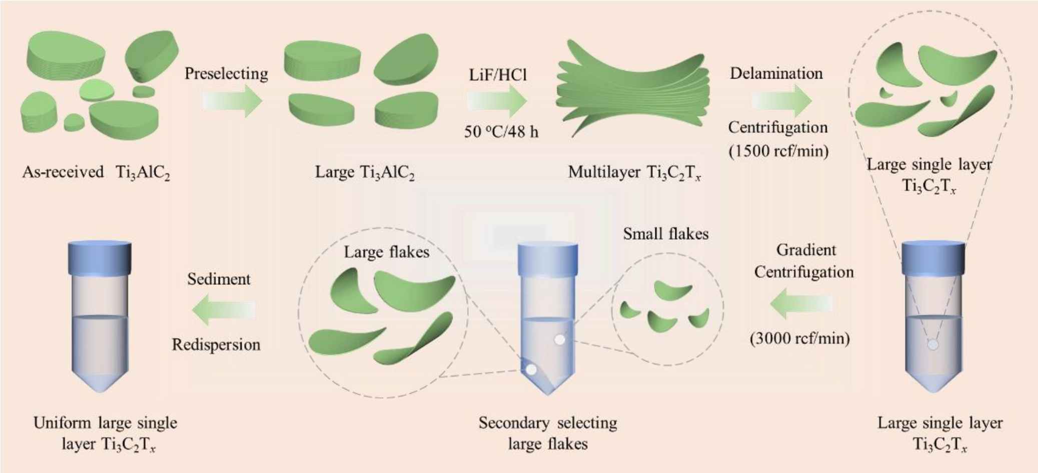


**Scheme S1** Schematic illustration of the synthesis process of ultralarge sized $Ti_3C_2T_x$ flakes from the as-received $Ti_3AlC_2$ phase.

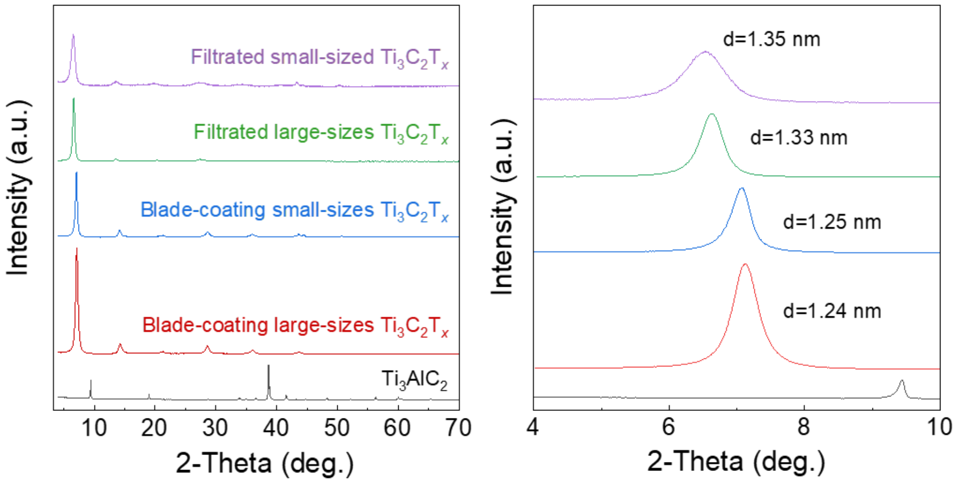


**Figure S1** XRD patterns of $Ti_3AlC_2$, opaque $Ti_3C_2T_x$ films with large-sizes and small-sizes by vacuum-assisted filtration and blade coating.

$Ti_3C_2T_x$ films prepared by blade coating have a smaller interlayer spacing than those of filtrated films. Their interlayer spacing is 1.24 nm (blade-coating large-sized $Ti_3C_2T_x$), 1.25 nm (blade-coating small-sized $Ti_3C_2T_x$), 1.33 nm (filtrated large-sized $Ti_3C_2T_x$), 1.35 nm (filtrated large-sizes $Ti_3C_2T_x$), respectively.

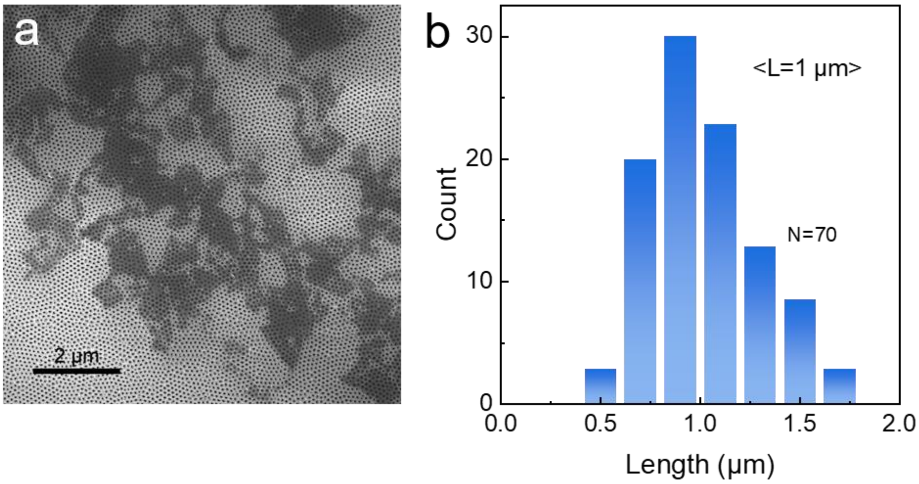


**Figure S2** **a** SEM image of small-sized $Ti_3C_2T_x$ flakes on anodic aluminum oxide (AAO). **b** Histogram of the small-sized $Ti_3C_2T_x$ flakes determined by statistical analysis.

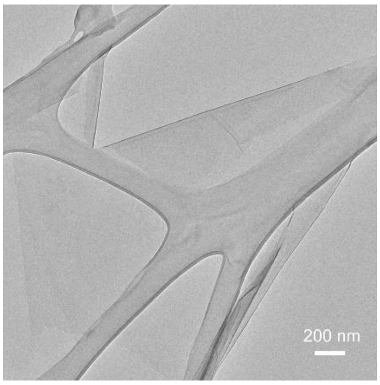


**Figure S3** TEM image of a $Ti_3C_2T_x$ flake

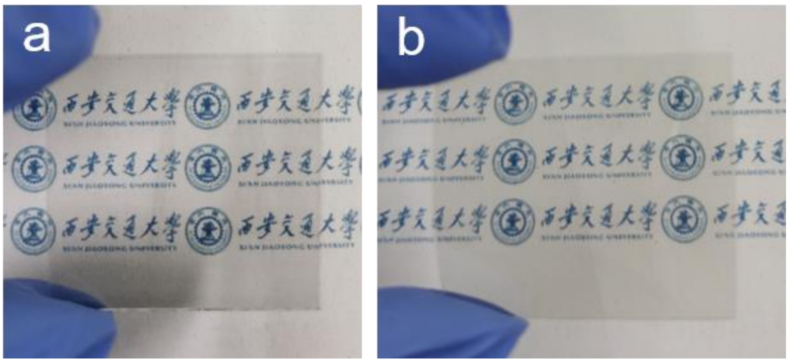


**Figure S4** Digital photograph of $Ti_3C_2T_x$ films from large-sizes flakes on glass (**a**, *T*=87%), on PET (**b**, *T*=91%).

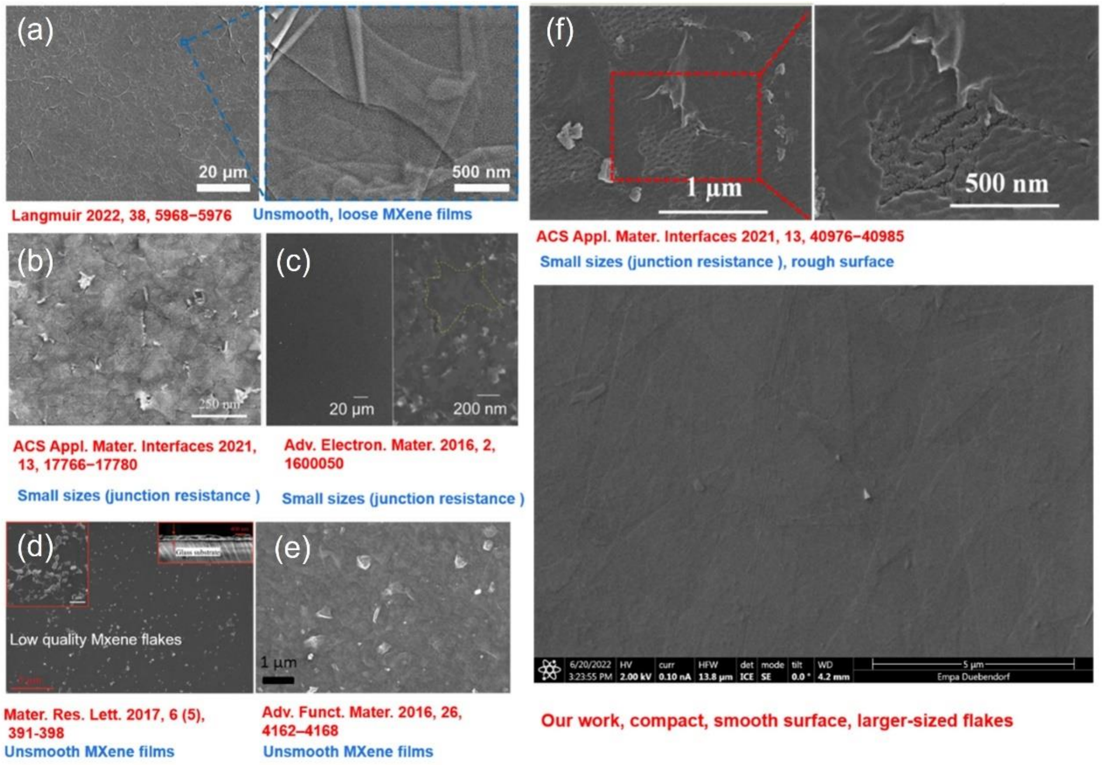


**Figure S5** Comparison of SEM images from previously published works with our transparent MXene film. **a** Reproduced with permission.[1] Copyright 2022, American Chemical Society. **b** Reproduced with permission.[2] Copyright 2021, American Chemical Society. **c** Reproduced with permission.[3] Copyright 2016, John Wiley and Sons. **d** Reproduced with permission.[4] Copyright 2017, Taylor & Francis Group. **e** Reproduced with permission.[5] Copyright 2016, John Wiley and Sons. **f** Reproduced with permission.[6] Copyright 2021, American Chemical Society.

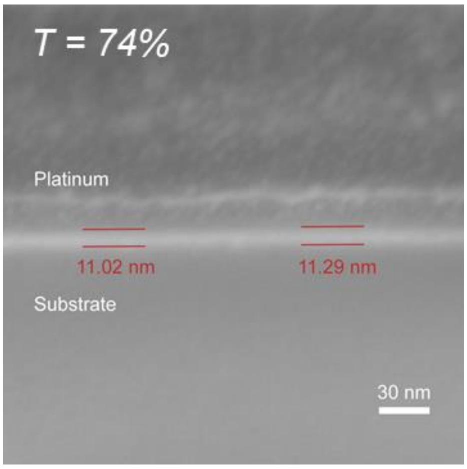


**Figure S6** Cross-sectional FIB-SEM image of a blade coated $Ti_3C_2T_x$ film. ($T$=74%).

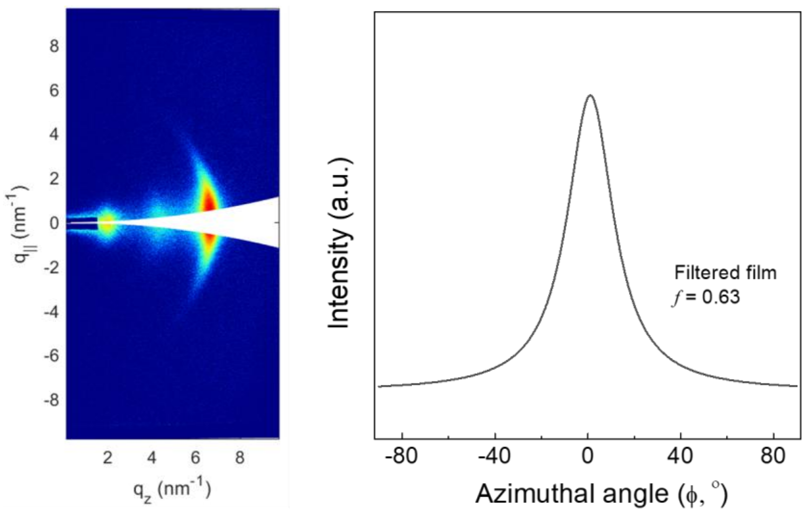


**Figure S7** GISAXS measurement of a filtered film (~1 μm). GISAXS detector image showing the (002) peak over $q_z$. Lorentzian fit of the azimuthal profile for the (002) peak used to determine Herman's degree of orientation.

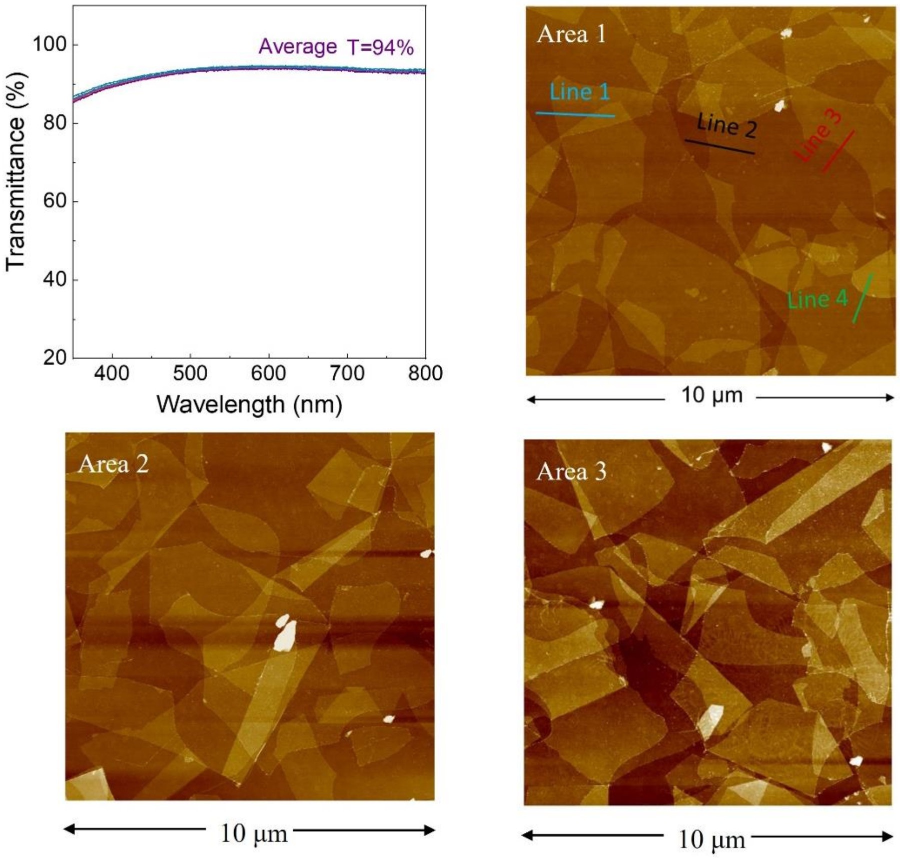


**Figure S8** The average transmittance of TCEs and the surface topography of different regions were measured by AFM.

Methods for calculating the number of layers of high transparency films ($T$=94%), according to the area percentage of the different layers $Ti_3C_2T_x$ flakes in the 10×10 μm area (for the area 1 image). Areas comprising different numbers of layers in Figure 3f are shown with different colors.

Table S1 Calculation of thickness of high transparency film ($T$=94%).

| | Pixel | Percentage (%) | Percentage (%) |
|---|---|---|---|
| Single layer | 371 691 | 37.5% | 35.7% |
| Double layers | 495 421 | 50.0% | 47.6% |
| Triple layers | 132 210 | 13.4% | 12.7% |
| Quadruple layers | 15 827 | 1.6% | 1.5% |
| Substrate (not covered) | 25 060 | 2.5% | 2.4% |
| Actual image pixel | 990 024 | 105% | |
| Statistical pixel totals | 1 040 209 | | 99.9% |

Area 1, image pixels: 996×994=990 024

The actual number of pixels counted according to different colors: 1 040 209

Thanks to the pixels at different color boundaries may be counted multiple times, the actual count is slightly higher than the number of pixels in the image.

Method 1:

Single layer: 371 691/990 024 =37.5%

Double layers: 495 421/990 024 =50.0%

Triple layers: 132 210/990 024 =13.4%

Quadruple layers: 15 827/990 024 =1.6%

Substrate: 25 060/990 024 = 2.5%

Layers calculation: 1×0.375+2×0.5+3×0.134+4×0.016=1.84

Percentage of coverage: 100%-2.5%=97.5%

Method 2:

Single layer: 371 691/1 040 209 =35.7%

Double layers: 495 421/1 040 209 =47.6%

Triple layers: 132 210/1 040 209 =12.7%

Quadruple layers: 15 827/1 040 209 =1.5%

Substrate: 25 060/1 040 209 = 2.4%

Layers calculation: 1×0.357+2×0.476+3×0.127+4×0.015=1.75

Percentage of coverage: 100%-2.4%=97.6%

Thus, the thickness of the TCE ($T$=94%) is 1.8 layers and the coverage is about 98%.

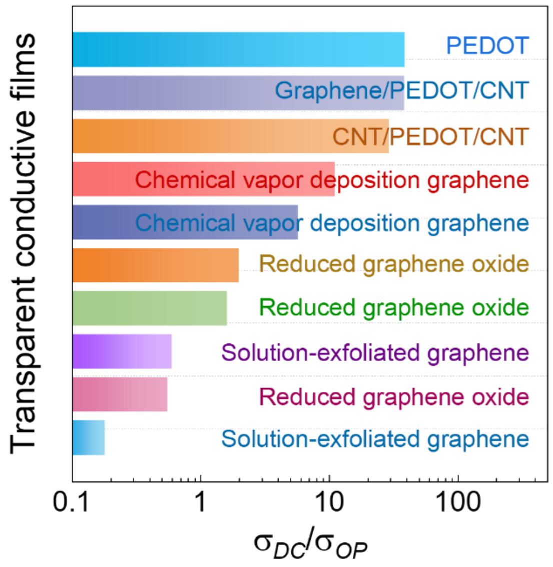


**Figure S9** Comparison of $\sigma_{DC}/\sigma_{op}$ ($FOM_e$) of various transparent conductive films, detailed values are presented in Table S2.

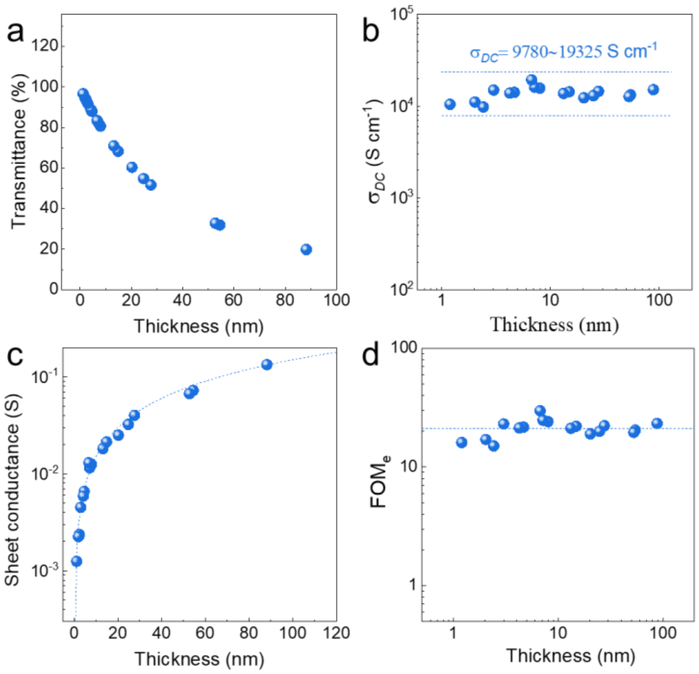


**Figure S10** Relationship between $Ti_3C_2T_x$ films thickness and optoelectronic properties. **a** Transmittance, **b** DC conductivity, **c** sheet conductance, **d** Ratio of DC conductivity to optical conductivity.

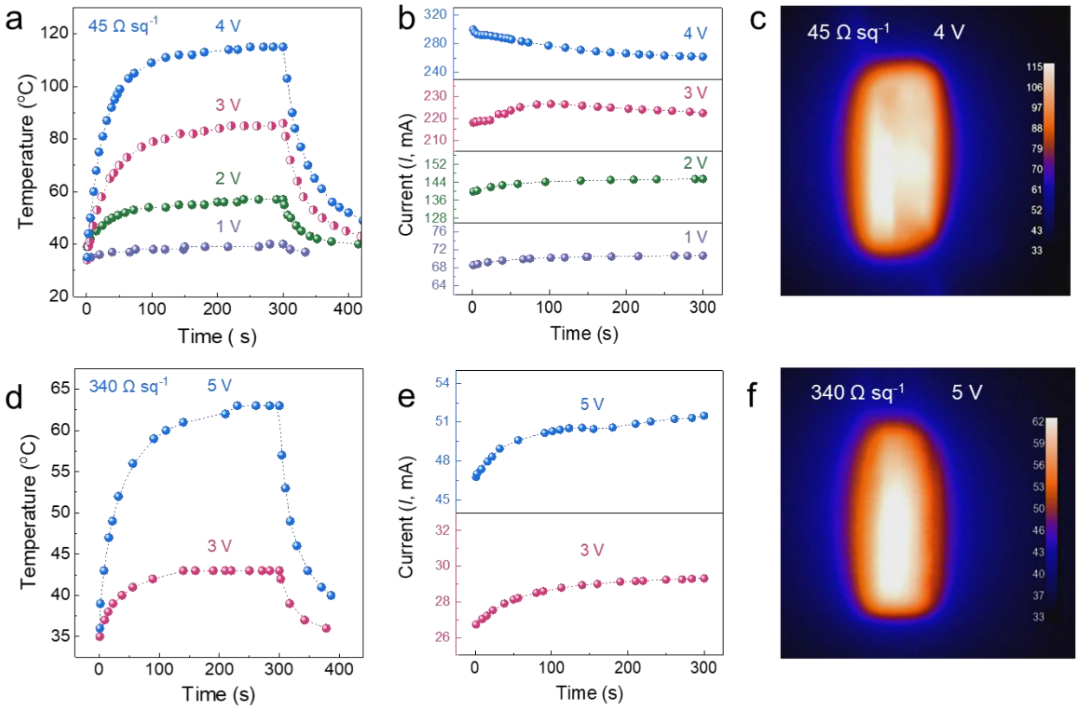


**Figure S11** Electrothermal properties of $Ti_3C_2T_x$ films. The time-varying surface temperature of the $Ti_3C_2T_x$ films with 45 Ω $sq^{-1}$ **a)** and 340 Ω $sq^{-1}$ **d)** for different applied voltages. **b, e)** The corresponding current values as a function of time. Infrared radiation (IR) images of the transparent $Ti_3C_2T_x$ films with 45 Ω $sq^{-1}$ **c)** and 340 Ω $sq^{-1}$ **f)**.

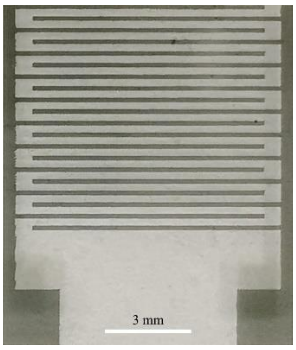


**Figure S12** Digital photograph of $Ti_3C_2T_x$-based symmetric micro-supercapacitors

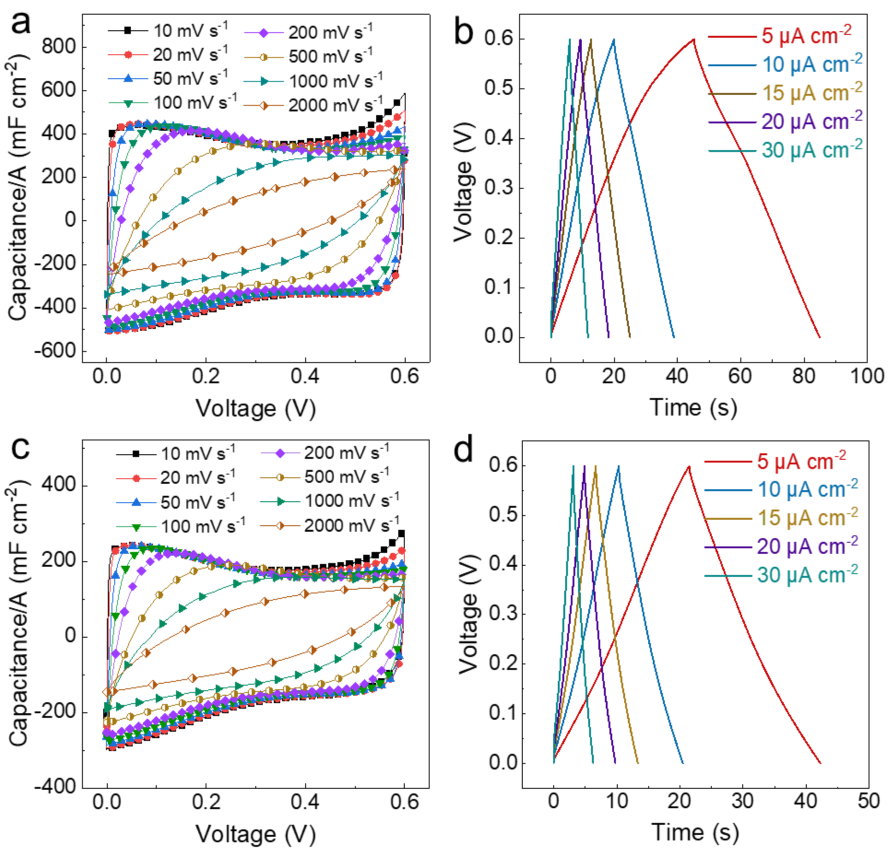


**Figure S13** Electrochemical characterization of $Ti_3C_2T_x$ films. Normalized CV curves at various scan rates of **a)** $T$=49%, **c)** T=61%, and GCD curves at different current densities of **b)** T=49%, **d)** T=61%.

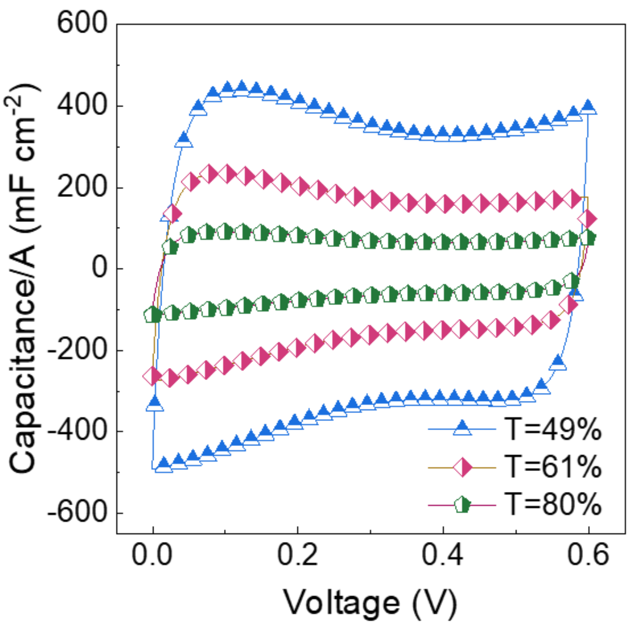


**Figure S14** CV curves with different transparency at 100 mV $s^{-1}$.

**Table S2** of **Figure 3b**

| Samples | Sizes | Manufacturing technique | Transmittance ($T_{550\ nm}$,%) | Sheet resistance ($\Omega\ sq^{-1}$) | $FoM_e$ | Ref. |
|---|---|---|---|---|---|---|
| $Ti_3C_2T_x$ | 100~300 nm | Inkjet-printing | 24 | 1500 | 0.12 | 2 |
| $Ti_3C_2T_x$ | ~500 nm | Spray-coating | ~51<br>~81 | ~625<br>~8160 | 0.51 | 3 |
| $Ti_3C_2T_x$ | ~80 nm | Spin-coating | 72<br>83<br>90<br>94 | 2010<br>3850<br>11870<br>23660 | 2 | 7 |
| $Ti_3C_2T_x$ | ~110 nm | Spin-coating | ~72<br>~87 | ~440<br>~8900 | 3.1 | 8 |
| $Ti_2CT_x$ | ~1 µm | Spin-coating | 65<br>80<br>86<br>96 | 128<br>507<br>1100<br>6440 | 5 | 4 |
| $V_2CT_x$ | 0.5~1 µm | Spin-coating | ~50 | ~67 | 6.5 | 9 |
| $Ti_3C_2T_x$ | — | Spin-coating | 86 | 330 | 7.3 | 5 |
| $Ti_3C_2T_x$ | ~0.5 µm | Dip-coating | ~86 | ~375 | 9 | 10 |
| $Ti_3C_2T_x$ | ~1.4 µm | Dip-coating | ~88 | ~600 | 14 | |
| $Ti_3C_2T_x$ | ~1.4 µm/ Optimizated | Dip-coating | ~89<br>~92 | ~320<br>~1870 | 17 | |
| $Ti_3C_2T_x$ | 1~2 µm | Dip-coating | 51<br>94 | 40<br>4300 | 14 | 11 |
| $Ti_3C_2T_x$ | ~3.2 µm | Spin-coating | 86<br>90<br>94 | 200<br>532<br>1031 | 15 | 12 |
| $Ti_3C_2T_x$ | 12.2 µm | Blade-coating | 20<br>60<br>81<br>89<br>94 | 7.5<br>40<br>80<br>170<br>423 | 21 | This work |

**Table S3** of **Figure S9**

| Samples | $FoM_e$ | Ref. |
|---|---|---|
| PEDOT | 39 | 13 |
| Graphene/PEDOT/CNT | 38.6 | 14 |
| CNT/PEDOT/CNT | 29.18 | 15 |
| CVD-graphene | 11.13 | 16 |
| CVD-graphene | 5.7 | 17 |
| Reduced graphene oxide | 2 | 18 |
| Reduced graphene oxide | 1.6 | 19 |
| Solution-exfoliate graphene | 0.6 | 20 |

| | | |
|---|---|---|
| Reduced graphene oxide | 0.55 | 21 |
| Solution-exfoliated graphene | 0.18 | 22 |

**Table S4** of **Figure 4c**

| Samples | Average electronic conductivity (S $cm^{-1}$) | Ref. |
|---|---|---|
| Reduced graphene oxide | 550 | 23 |
| Reduced graphene oxide | 1425 | 24 |
| $Ti_3C_2T_x$ | 3092 | 8 |
| $Ti_2CT_x$ | 5250 | 4 |
| $Ti_3C_2T_x$ | 6500 | 5 |
| $Ti_3C_2T_x$ | 7450 | 12 |
| $Ti_3C_2T_x$ | 7530 | 10 |
| $Ti_3C_2T_x$ | 15000 | This work |

**Table S5** of **Figure 6g**

| Samples | Transmittance ($T_{550}$ nm,%) | Capacitance (μF $cm^{-2}$) | Ref. |
|---|---|---|---|
| Graphene Q-dot | 93 | 9.1 | 25 |
| Graphene networks | 84 | 4.2 | 26 |
| Wrinkled graphene | 60 | 5.8 | 27 |
| Graphene film | 67 | 12.4 | 28 |
| $Ti_3C_2T_x$ | 88 | 19 | 11 |
| | 38 | 283 | |
| $Ti_3C_2T_x$ | 81 | 870 | 12 |
| $Ti_3C_2T_x$ | 73 | 192 | 2 |
| $Ti_3C_2T_x$ | 49 | 383.16 | This work |
| | 61 | 189.5 | |
| | 80 | 75.3 | |

**Table S6** of **Figure 6h**

| Samples | Transmittance ($T_{550}$ nm, %) | Power density ($\mu$W cm$^{-2}$) | Energy density($\mu$Wh cm$^{-2}$) | Ref. |
|---|---|---|---|---|
| Graphene film | 67 | 70 | 0.00047 | 28 |
| $Ti_3C_2T_x$ | 88 | 0.0188 | 0.00163 | 11 |
| | 38 | 1.3 | 0.01 | |
| $Ti_3C_2T_x$ | 73 | 0.077 | 0.0043 | 2 |
| $Ti_3C_2T_x$ | 49 | 1.1496 | 0.01916 | This work |
| | | 2.28 | 0.019 | |
| | | 5.526 | 0.01842 | |
| | | 10.608 | 0.01768 | |
| | | 19.836 | 0.01653 | |
| | 61 | 0.5682 | 0.00947 | |
| | | 1.1244 | 0.00937 | |
| | | 2.733 | 0.00911 | |
| | | 5.244 | 0.00874 | |
| | | 9.792 | 0.00816 | |
| | 80 | 0.2256 | 0.00376 | |
| | | 0.4512 | 0.00376 | |
| | | 1.098 | 0.00366 | |
| | | 2.118 | 0.00353 | |
| | | 3.948 | 0.00329 | |

**References**


(1) Huang, L.; Lin, Y.; Zeng, W.; Xu, C.; Chen, Z.; Wang, Q.; Zhou, H.; Yu, Q.; Zhao, B.; Ruan, L.; et al. Highly Transparent and Flexible Zn-$Ti_3C_2T_x$ MXene Hybrid Capacitors. *Langmuir* **2022**, *38*, 5968-5976.

(2) Wen, D.; Wang, X.; Liu, L.; Hu, C.; Sun, C.; Wu, Y.; Zhao, Y.; Zhang, J.; Liu, X.; Ying, G. Inkjet Printing Transparent and Conductive MXene ($Ti_3C_2T_x$) Films: A Strategy for Flexible Energy Storage Devices. *ACS Appl. Mater. Inter.* **2021**, *13*, 17766-17780.

(3) Hantanasirisakul, K.; Zhao, M. Q.; Urbankowski, P.; Halim, J.; Anasori, B.; Kota, S.; Ren, C. E.; Barsoum, M. W.; Gogotsi, Y. Fabrication of $Ti_3C_2T_x$ MXene Transparent

Thin Films with Tunable Optoelectronic Properties. *Adv. Electron. Mater.* **2016**, *2*, 1600050.

(4) Ying, G.; Dillon, A. D.; Fafarman, A. T.; Barsoum, M. W. Transparent, conductive solution processed spincast 2D $Ti_2CT_x$ (MXene) films. *Mater. Res.h Lett.* **2017**, *5*, 391-398.

(5) Dillon, A. D.; Ghidiu, M. J.; Krick, A. L.; Griggs, J.; May, S. J.; Gogotsi, Y.; Barsoum, M. W.; Fafarman, A. T. Highly Conductive Optical Quality Solution-Processed Films of 2D Titanium Carbide. *Adv. Funct. Mater.* **2016**, *26*, 4162-4168.

(6) Kumar, S.; Kang, D.; Nguyen, V. H.; Nasir, N.; Hong, H.; Kim, M.; Nguyen, D. C.; Lee, Y. J.; Lee, N.; Seo, Y. Application of Titanium-Carbide MXene-Based Transparent Conducting Electrodes in Flexible Smart Windows. *ACS Appl. Mater. Inter.* **2021**, *13*, 40976-40985.

(7) Ebrahimi, M.; Mei, C. T. Optoelectronic properties of $Ti_3C_2T_x$ MXene transparent conductive electrodes: Microwave synthesis of parent MAX phase. *Ceram. Int.* **2020**, *46*, 28114-28119.

(8) Mariano, M.; Mashtalir, O.; Antonio, F. Q.; Ryu, W. H.; Deng, B.; Xia, F.; Gogotsi, Y.; Taylor, A. D. Solution-processed titanium carbide MXene films examined as highly transparent conductors. *Nanoscale* **2016**, *8*, 16371-16378.

(9) Ying, G.; Kota, S.; Dillon, A. D.; Fafarman, A. T.; Barsoum, M. W. Conductive transparent $V_2CT_x$ (MXene) films. *FlatChem* **2018**, *8*, 25-30.

(10) Salles, P.; Pinto, D.; Hantanasirisakul, K.; Maleski, K.; Shuck, C. E.; Gogotsi, Y. Electrochromic Effect in Titanium Carbide MXene Thin Films Produced by Dip-Coating. *Adv. Funct. Mater.* **2019**, *29*, 1809223.

(11) Salles, P.; Quain, E.; Kurra, N.; Sarycheva, A.; Gogotsi, Y. Automated Scalpel Patterning of Solution Processed Thin Films for Fabrication of Transparent MXene Microsupercapacitors. *Small* **2018**, *14*, e1802864.

(12) Zhang, C. J.; Anasori, B.; Seral-Ascaso, A.; Park, S. H.; McEvoy, N.; Shmeliov, A.; Duesberg, G. S.; Coleman, J. N.; Gogotsi, Y.; Nicolosi, V. Transparent, Flexible, and Conductive 2D Titanium Carbide (MXene) Films with High Volumetric Capacitance. *Adv. Mater.* **2017**, *29*, 1702678.

(13) Higgins, T. M.; Coleman, J. N. Avoiding Resistance Limitations in High-Performance Transparent Supercapacitor Electrodes Based on Large-Area, High-Conductivity PEDOT:PSS Films. *ACS Appl. Mater. Inter.* **2015**, *7*, 16495-16506.

(14) Wang, T.; Wang, Y. Z.; Jing, L. C.; Zhu, Q.; Ethiraj, A. S.; Geng, W.; Tian, Y.; Zhu, Z.; Meng, Z.; Geng, H. Z. Novel biodegradable and ultra-flexible transparent conductive film for green light OLED devices. *Carbon* **2021**, *172*, 379-389.

(15) Gu, Z. Z.; Tian, Y.; Geng, H. Z.; Rhen, D. S.; Ethiraj, A. S.; Zhang, X.; Jing, L. C.; Wang, T.; Xu, Z. H.; Yuan, X. T. Highly conductive sandwich-structured CNT/PEDOT:PSS/CNT transparent conductive films for OLED electrodes. *Appl. Nanosci.* **2019**, *9*, 1971-1979.

(16) Cai, W.; Zhu, Y.; Li, X.; Piner, R. D.; Ruoff, R. S. Large area few-layer graphene/graphite films as transparent thin conducting electrodes. *Appl. Phys. Lett.* **2009**, *95*, 123115.

(17) Kim, K. S.; Zhao, Y.; Jang, H.; Lee, S. Y.; Kim, J. M.; Kim, K. S.; Ahn, J. H.; Kim, P.; Choi, J. Y.; Hong, B. H. Large-scale pattern growth of graphene films for stretchable transparent electrodes. *Nature* **2009**, *457*, 706-710.

(18) Zhao, J. P.; Pei, S. F.; Ren, W. C.; Gao, L. B.; Cheng, H. M. Efficient Preparation of Large-Area Graphene Oxide Sheets for Transparent Conductive Films. *ACS Nano* **2010**, *4*, 5245-5252.

(19) Becerril, H. A.; Mao, J.; Liu, Z.; Stoltenberg, R. M.; Bao, Z.; Chen, Y. Evaluation of solution-processed reduced graphene oxide films as transparent conductors. *ACS Nano* **2008**, *2*, 463-470.

(20) Green, A. A.; Hersam, M. C. Solution Phase Production of Graphene with Controlled Thickness via Density Differentiation. *Nano Lett.* **2009**, *9*, 4031-4036.

(21) Liang, Y.; Frisch, J.; Zhi, L.; Norouzi-Arasi, H.; Feng, X.; Rabe, J. P.; Koch, N.; Mullen, K. Transparent, highly conductive graphene electrodes from acetylene-assisted thermolysis of graphite oxide sheets and nanographene molecules. *Nanotechnology* **2009**, *20*, 434007.

(22) Li, X. L.; Zhang, G. Y.; Bai, X. D.; Sun, X. M.; Wang, X. R.; Wang, E.; Dai, H. J. Highly conducting graphene sheets and Langmuir-Blodgett films. *Nat. Nanotechnol.* **2008**, *3*, 538-542.

(23) Wang, X.; Zhi, L. J.; Mullen, K. Transparent, conductive graphene electrodes for dye-sensitized solar cells. *Nano Lett.* **2008**, *8*, 323-327.

(24) Liang, Y. Y.; Frisch, J.; Zhi, L. J.; Norouzi-Arasi, H.; Feng, X. L.; Rabe, J. P.; Koch, N.; Mullen, K. Transparent, highly conductive graphene electrodes from acetylene-assisted thermolysis of graphite oxide sheets and nanographene molecules. *Nanotechnology* **2009**, *20*.

(25) Lee, K.; Lee, H.; Shin, Y.; Yoon, Y.; Kim, D.; Lee, H. Highly transparent and flexible supercapacitors using graphene-graphene quantum dots chelate. *Nano Energy* **2016**, *26*, 746-754.

(26) Fan, X.; Chen, T.; Dai, L. Graphene networks for high-performance flexible and transparent supercapacitors. *RSC Adv.* **2014**, *4*, 36996.

(27) Chen, T.; Xue, Y. H.; Roy, A. K.; Dai, L. M. Transparent and Stretchable High-Performance Supercapacitors Based on Wrinkled Graphene Electrodes. *ACS Nano* **2014**, *8*, 1039-1046.

(28) Gao, Y.; Zhou, Y. S.; Xiong, W.; Jiang, L. J.; Mahjouri-samani, M.; Thirugnanam, P.; Huang, X.; Wang, M. M.; Jiang, L.; Lu, Y. F. Transparent, flexible, and solid-state supercapacitors based on graphene electrodes. *APL Mater.* **2013**, *1*, 012101.
